# From an Unfolding Emergency Treatment to a Universal Shift in Therapy: The History and Evolution of the Catheter-Based Treatment of Aortic Valve Stenosis

**DOI:** 10.31083/j.rcm2310349

**Published:** 2022-10-17

**Authors:** Hüseyin Umut Agma, Alexandros Krull, Sebastian Feickert, Raid Al Ammareen, Hüseyin Ince, Giuseppe D’Ancona

**Affiliations:** ^1^Department of Cardiology, Internal Medicine and Conservative Intensive Care Medicine, Vivantes Klinikum Am Urban, 10967 Berlin, Germany; ^2^Department of Cardiology, Internal Medicine and Conservative Intensive Care Medicine, Vivantes Klinikum Neukölln, 12351 Berlin, Germany; ^3^Department of Cardiology, Rostock University Medical Center, 18057 Rostock, Germany

**Keywords:** TAVI, prosthesis, aortic, valve, stenosis

## Abstract

Aortic valve stenosis (AVS) is the most frequent valvular heart disease in industrialized countries, presenting with very high mortality if left untreated. While drug treatment can sometimes alleviate symptoms, it fails to stop progression or cure the underlying disease. Until the first decade of this millennium, surgical aortic valve replacement (SAVR) remained the only available therapy option with a positive impact on mortality and morbidity. Even though several studies reported highly positive effects of SAVR regarding the improved quality of life and better physical performance, SAVR remained an intervention that, due to its remarkable complexity and the need for heart-lung machine and cardioplegia, was limited by the patients’ comorbid profile. While unsatisfying hemodynamic results after transcatheter aortic balloon valvuloplasty in high-risk surgical patients limited its adoption as an alternative treatment, it provided the impetus for further interventional approaches to the therapy of AVS. This review considers the invention and development of transcatheter aortic valve implantation (TAVI), which established itself as a catheter-based, minimally invasive procedure over the past decade, and has become an equivalent treatment method for high-risk surgical patients. For that matter, early TAVI concepts, their amendments, and the associated pioneers are recognized for paving the way to a revolutionary diversification in AVS treatment.

## 1. Epidemiology

Aortic valve stenosis (AVS) describes a narrowing of the aortic valve, which usually occurs due to calcification of a congenital bicuspid or tricuspid valve or as a result of rheumatic disease, is the most common valvular pathology requiring treatment in Europe and North America [1]. The prevalence of AVS increases with the age of patients. In the age group of 50- to 59-year-old patients, the prevalence is 0.2%, whereas in the 80- to 89-year-old, the prevalence is already at 9.8% [2]. According to a meta-analysis of 7 studies, the pooled prevalence of AVS in patients >75 years is 3.4% in Europe and North America [3]. Thus, it is estimated that approximately 290,000 patients worldwide with high-grade AVS would be potential candidates for transcatheter aortic valve implantation (TAVI), and approximately 27,000 new patients are added every year [3].

## 2. Etiology and Classification 

The classification of AVS is based either on etiology or severity. Etiologically it is to differ between a congenital, rheumatic, or senile AVS [4], with the senile AVS being the most common cause of AVS in Europe and North America [5]. However, the classification according to severity is more decisive for the necessity of therapy. According to the latest American [6] and European [7] guidelines, the severity of AVS is usually determined noninvasively by Doppler echocardiography and defined as depicted in Table 1.

**Table 1.** **Current Guidelines for the Management of AVS—Categories of severe AVS based on the 2020 ACC/AHA Guidelines and 2021 ESC/EACTS Guidelines**.

2020 ACC/AHA Guidelines for the Management of Patients With Valvular Heart Disease		2021 ESC/EACTS Guidelines for the Management of Patients With Valvular Heart Disease	
Stage	Valve hemodynamics	Stage	Valve hemodynamics
Stage A	At risk of AVS with peak velocity <2.0 m/s	Not described	
Stage B	Mild AVS, mean gradient <20 mmHg, or peak velocity 2.0–2.9 m/s	Not described	
	Moderate AVS, mean gradient 20–39 mmHg, or peak velocity 3.0–3.9 m/s	Normal-flow, low-gradient AVS with preserved ejection fraction	Usually, moderate AVS, mean gradient <40 mmHg AVA ≤1 ${\mathrm{cm}}^{2}$, LVEF ≥50%, or SVi >35 mL/$m^{2}$
Stage C1, Asymptomatic severe AVS with preserved ejection fraction	mean gradient ≥40 mmHg	High-gradient AVS	mean gradient ≥40 mmHg, peak velocity ≥4.0 m/s, AVA ≤1 ${\mathrm{cm}}^{2}$, or AVAi ≤0.6 ${\mathrm{cm}}^{2}$/$m^{2}$
	peak velocity ≥4.0 m/s		
	AVA is not required		
	LVEF ≥50%		
Stage C2, Asymptomatic severe AVS with reduced ejection fraction	mean gradient ≥40 mmHg		
	peak velocity ≥4.0 m/s		
	AVA is not required		
	LVEF ≤50%		
Stage D1, Symptomatic severe high-gradient AVS	mean gradient ≥40 mmHg		
	peak velocity ≥4.0 m/s		
	AVA is not required		
	LVEF ≥50%		
Stage D2, Symptomatic severe low-flow, low-gradient AVS with reduced LVEF	mean gradient <40 mmHg	Low-flow, low-gradient AVS with reduced ejection fraction	mean gradient <40 mmHg, peak velocity <4.0 m/s, AVA ≤1 ${\mathrm{cm}}^{2}$, LVEF <50%, or ≤35 mL/m${\mathrm{SVi}}^{2}$
	peak velocity <4.0 m/s		
	AVA ≤1 ${\mathrm{cm}}^{2}$		
	LVEF <50%		
Stage D3, Symptomatic severe AVS with normal LVEF or paradoxical low-flow severe AVS	mean gradient <40 mmHg	Low-flow, low-gradient aortic stenosis with preserved ejection fraction	mean gradient <40 mmHg, peak velocity <4.0 m/s, AVA ≤1 ${\mathrm{cm}}^{2}$, LVEF ≥50%, SVi ≤35 mL/$m^{2}$
	peak velocity <4.0 m/s		
	AVA ≤1 ${\mathrm{cm}}^{2}$		
	AVAi ≤0.6 ${\mathrm{cm}}^{2}$/$m^{2}$		
	LVEF ≥50%		
	SVi ≤35 mL/$m^{2}$		

This table gives a brief overview about the main differences and similarities with, without claims of completeness of information. For detailed information the underlying guidelines should be read. Abbreviations, ACC, American Collage of Cardiology; AHA, American Heart Association; ESC, European Society of Cardiology; EACTS, European Association for Cardio-Thoracic Surgery; AVA, Aortic Valve Area; AVAi, indexed Aortic Valve Area; LVEF, left ventricular ejection fraction; SVi, indexed stroke volume.

Whereas the European Society of Cardiology (ESC) guidelines define four significant categories of severe AVS based on the peak flow, gradient and left ventricular ejection fraction (LVEF), the American guidelines categorize the severe AVS into stages mainly based on symptoms followed by the parameters mentioned above, leading to a much more detailed subdivision of AVS. The American guidelines also consider more patient-relevant nuances for the following decision-making process for the ideal therapy of AVS. Table 1 shows the different stages of AVS according to European and American guidelines.

## 3. Pathophysiology 

The pathophysiology of AVS differs depending on the underlying etiology. The senile or calcified AVS is a chronic progressive process affecting mainly older patients. Like coronary artery disease, calcification and an inflammatory reaction of the valve leaflets occur, resulting in a stiffening of the leaflets, evoking a progressive narrowing of the valve’s lumen [8]. At this moment, higher ventricular pressure is needed to sustain the required blood flow across the aortic valve (AV), leading to a pressure overload and ultimately to left ventricular hypertrophy and heart failure [9]. The course of this type of AVS is chronically progressive. It has a high mortality of approximately 25% per year, with an average survival of two to three years once it gets symptomatic and stays untreated [10].

The bicuspid AV is a congenital malformation occurring sporadically due to an autosomal dominant inheritance with incomplete penetrance [11, 12, 13]. It is the most common clinically relevant congenital heart defect, after a ventricular septal defect and a persistent foramen ovalis, occurring in about 1% to 2% of the population [14, 15, 16]. In this case, the AV consists of only two leaflets. Two of the naturally three leaflets are usually fused, with a high variety of the number of commissures and the presence of a raphe [16]. Due to increased mechanical stress, a degenerative remodeling process leads to the calcification of the valve [12]. The AVS starts to get symptomatic in patients with bicuspid AV about 20 years earlier than in patients with severe AVS and tricuspid AV [9].

The last form of AVS is rheumatic AVS. It occurs as a result of rheumatic fever following bacterial infection with β-hemolytic streptococci and is most commonly experienced in the setting of pharyngitis [17]. In this case, the formation of autoantibodies against cardiac structures leads to endocarditis or pericarditis, possibly causing scarring of the mitral and aortic valves in particular [18, 19]. However, this type of AVS has become a rarity in North America and Europe due to available antibiotic treatment for streptococcal infection. Despite the different etiopathogenesis, all three forms of AVS result in a chronic pressure overload of the left ventricle with a consecutive left ventricular hypertrophy and, in the further course, the development of left heart failure.

## 4. Symptoms

AVS remains asymptomatic for a long time, especially if patients unconsciously are not as active as they used to be and do not exercise in general. The leading symptoms in high-grade AVS are dyspnea, angina pectoris, and dizziness or syncope during or after exertion [6, 20].

## 5. Therapy

No drug treatment is available for severe AVS at this juncture. Thus, surgical aortic valve replacement (SAVR) or TAVI is the therapy of choice [4, 7], the latter being a therapeutic option increasing worldwide in the last two decades. The third option of treatment is a simple balloon valvuloplasty of the AVS. However, this procedure is usually performed only as a palliative therapy or as a bridge to decision and treatment in patients who need urgent therapy and are not yet suitable for SAVR or TAVI [21].

**Fig. 1.**
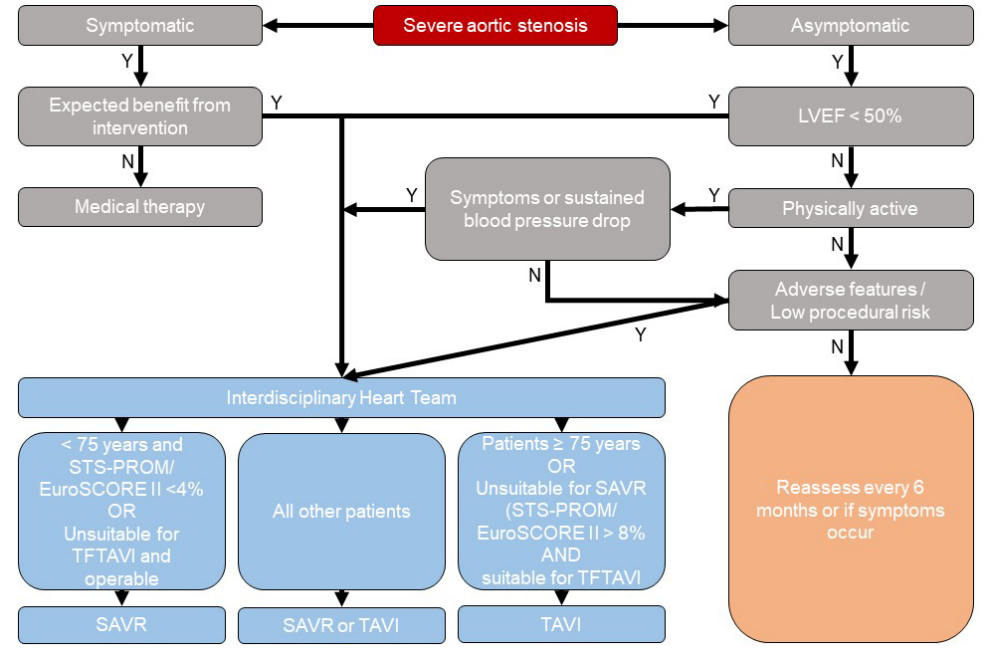
**Management of patients with severe aortic stenosis based on the 2021 ESC/EACTS Guidelines**. LVEF, left ventricular ejection fraction; SAVR, surgical aortic valve replacement; TAVI, transcatheter aortic valve implantation.

Following the latest American and European guidelines for the management and treatment of patients with AVS, the indication for treatment of severe AVS is usually given at the point when the previously mentioned symptoms first develop. All patients are discussed in a multidisciplinary heart team to decide whether SAVR or TAVI is the most suitable therapy option. Fig. 1 shows the workflow of decision-making and therapy evaluation in patients with severe AVS according to the European guidelines. Usually, patients younger than 75 with a low risk for SAVR (STS-PROM/EuroScore II <4%) are suggested to undergo a SAVR. On the other hand, patients 75 years or older, and/or with high risk for SAVR (defined by a STS-PROM/EuroScore II >8%) and generally suitable for a transfemoral transcatheter procedure, are suggested to undergo TAVI [7]. The suitability for TAVI must keep into consideration the quality of the vascular access and the AV “landing-zone”. In this context, although bicuspid AVS is nowadays routinely treated with TAVI, patients with this condition should be evaluated thoroughly to document the possibility of achieving optimal prosthesis function after TAVI.

In the beginning, TAVI was only used as a palliative treatment for patients unsuitable for SAVR. Since the first success in humans was achieved by Cribier *et al*. [22] in 2002, TAVI has advanced to effective therapy of severe AVS, similar to the SAVR. Especially since the latest trials (Placement of Aortic Trans-Catheter Valve (PARTNER 3) and Surgical Replacement and Transcatheter Aortic Valve Implantation (SURTAVI, Evolut Low Risk), indication for TAVI from high-risk patients was also entrenched for low-risk patients [23, 24].

In the following paragraphs of this manuscript, we will summarize the extraordinary pioneering work done to develop the management of severe symptomatic AVS and bring TAVI to become the minimally invasive procedure it is today.

## 6. A Brief History of AVR

### 6.1 Valvuloplasty and Mechanical Prostheses

The early steps of SAVR can be traced to the 1940s with the introduction of the first mechanical aortic valve prosthesis by Charles Hufnagel, made of a methacrylate chamber containing a methacrylate ball. The prosthesis was implanted in the descending aorta to treat severe aortic valve regurgitation [25].

However, the first annular implantation of an aortic valve prosthesis required the existence of a reliable cardiopulmonary bypass (CPB, commonly known as a “heart-lung machine”) that emerged in the mid-1950s. This development allowed Dwight Harken to perform the very first true SAVR through the implantation of a “double caged ball” mechanical prosthesis called “Harken-Soroff” in 1960 [26].

Before this development, Harken was an avid supporter of transaortic valvuloplasty (through digital or instrumental dilatation of the native AV) in adult patients with calcific AVS [27]. This “closed procedure” did not require full aortotomy and was performed without using the heart-lung machine. Unfortunately, the procedure resulted in a concerning rate of severe iatrogenic AV regurgitation and a high rate of AVS recurrence [28].

The first mechanical prosthesis with a reduced profile was the Starr-Edwards caged-ball prosthesis in 1961 [29]. The disadvantages of caged ball prostheses ranged from minor inconveniences like valve noise to significant limitations like restricted effective orifice area (EOA), turbulent extra-centric flow, hemolysis, and the need for aggressive anticoagulation.

**Fig. 2.**
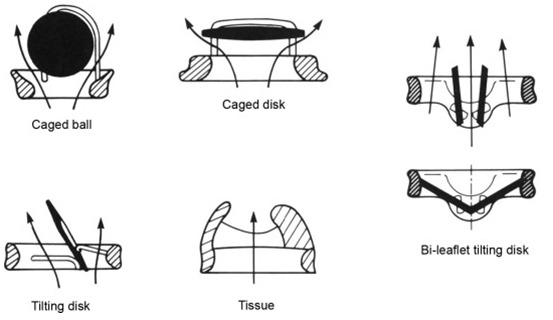
**“Designs and flow patterns of major categories of prosthetic heart valves: caged-ball, caged-disk, tilting-disk, bileaflet tilting-disk, and bioprosthetic (tissue) valves. Whereas flow in mechanical valves must course along both sides of the occluder, bioprostheses have a central flow pattern”. **Reproduced with permission from [30]—Copyright 1983, Pergamon Press Limited, 1983; and Copyright © 1985 Springer-Verlag, Inc.

The development of a tilting-disc prosthesis promised a less turbulent, still mildly eccentric flow. One of the early examples was the Björk-Shiley valve (Fig. 2, [30]), a spherical tilting-disc prosthesis featuring one major and one minor orifice and a tilting disc with two struts [31, 32]. Another example of a tilting-disc prosthesis was the Lillehei-Kaster valve [33]. Although modifications to this design tried to reduce the valvular resistance through a convexo-concave tilting disc, the valve was inherently prone to thrombus formation and excessive tissue overgrowth, especially around the minor orifice.

Kalke and Lillehei [34] developed the first rigid bi-leaflet valve, yet the first mass-production bi-leaflet valve prothesis was produced by St. Jude Medical (SJM). These “next-generation” valves were promised a more laminar flow and less blood stagnation. The lower profile led to easier implantation and better orientation for physiological flow. Even though the trend towards bi-leaflet valves as the preferred mechanical prosthesis persists to this day, no available data has shown a significant survival benefit favoring the bi-leaflet valves compared to the tilting-disc prostheses [35]. Sixty years after the first implantation, the latest generations of mechanical valves possess a better hemodynamic performance and reduced hemolysis. However, the current data remains inadequate for altering the standard anticoagulation regimen [6, 36, 37, 38, 39].

### 6.2 The Advent of Biological Valves

The history of the idea of AVR can be traced to the first homograft replacement of the AV, which was performed in 1962 by Donald Ross and published in Lancet [40, 41, 42, 43]. The Ross procedure defines the replacement of the AV with the patient’s pulmonary valve (autograft) and the replacement of the pulmonary valve by an aortic or pulmonary allograft.

The first xenograft biological valves were native porcine valves treated with glutaraldehyde, making them immunogenically inactive and preventing the denaturation process. The introduction of anti-calcification treatment at this point already contributed to the durability of the early valves. Carpentier [42] was the first to mount whole porcine valves into a stented 3-D structure which facilitated easier device implantation. However, this approach brought unanticipated limitations as the structure’s stiffness and limited range of movement of the porcine leaflet in native formation resulted in a suboptimal hemodynamical performance of the valve [42].

Owing to the improvements in xenograft tissue preparation and modification, the first pericardial prosthesis with a flexible stent was developed by Ionescu in 1971 [43]. Numerous modifications to the stent structure, leaflet material, and design resulted in progressive, more durable, and better-performing valves [43].

Another limitation of stented annular xenograft prostheses that emerged since the early stages of development of this technology was the risk of having a low prosthetic effective orifice area (EOA). In fact, in the presence of a small native aortic annular diameter, the prosthesis EOA was further reduced by the space occupied by the prosthesis circular stent and sewing ring and could result in “Patient-Prosthesis-Mismatch” (PPM) with persistent valvular gradients despite SAVR. PPM further increased the rate of structural valve deterioration (SVD) and overall risk of mortality [44].

The enthusiasm about stentless biological valves to overcome stented-valve PPM faded over time mainly because stentless bioprostheses did not seem to have a superior performance in long-term studies [45].

A surgical annuloplasty for aortic annular enlargement, to allow for implantation of a larger prosthesis and reduce the risk of PPM, was proposed as early as 1979 [46], but carried higher surgical complexity, hindering the chance of potential global adoption of the technique [47].

More recently, the desire for reduced invasiveness and a simplified procedure has led to the development of minimally invasive AVR with sutureless aortic valve prostheses where the valve stent has a very low profile and can achieve good annular fixation and sealing without surgical sutures [48, 49, 50, 51] (Table 2).

**Table 2.** **Commercially available sutureless aortic valve protheses**.

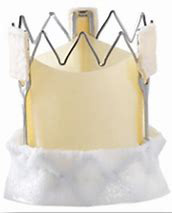	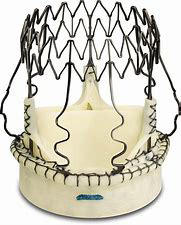	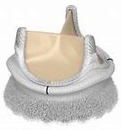
3F Enable (Medtronic, Minneapolis, MN, USA)	Perceval S (Sorin, Saluggia, Italy)	Intuity Elite (Edward Lifesciences, Irvine, CA, USA)

The role of these valves in high and intermediate-risk patients has been partly overtaken by the advent of TAVI [52].

The technological evolution of biologic AV prostheses (stent and leaflet tissue) and changes in the referral pattern for AVR in patients with AVS have facilitated their increasing adoption. The so-called “tissue valves” have proven an acceptable performance and durability without long-term anticoagulation [53]. These features make bioprostheses the best option for treating elderly patients, representing most AVR candidates. Mechanical valves are still selectively adopted in younger patients considering their extended life expectancy.

### 6.3 The Dawn of Percutaneous Intervention in Cardiovascular Medicine

The attempt to avoid the detrimental effects of the heart-lung machine and cardiac arrest to perform AVR has supported the development of a minimally invasive catheter-based and percutaneous approach to treating AVS.

The most evocative metaphor applied to scientific progress is building an edifice of knowledge, with every innovation and idea being a brick [54]. Following this allegory, we could identify Charles Dotter, the inventor of percutaneous transluminal angioplasty (PTA), as one of the first masons in the field of performing the first PTA in 1964 on a patient with severe peripheral artery disease (PAD) [55, 56]. Inspired by his lecture in Frankfurt the following year, Andreas Grüntzig [57] conceptualized and performed the first-in-human percutaneous transluminal coronary angioplasty (PTCA) in 1977 in Zurich. This remarkable chain of inspiration continued through Julio Palmaz. He was greatly inspired by the lecture of Grüntzig [57] in New Orleans in 1978 and eventually developed the first balloon-expandable coronary stent.

Henning Rud Andersen, who designed and performed the first-in-animal (FIA) TAVI, openly recalls getting his inspiration from Palmaz during a conference in Scottsdale, Arizona, the USA, in 1989 [55, 58].

### 6.4 Revisiting an Old Concept with New Techniques 

The use of percutaneous transcatheter balloon dilatation for valvuloplasty started in the early 1980s. Carl J. Pepine [59] published the first case report of a transvenous transcatheter valvuloplasty of the pulmonary valve in an adult patient in 1982. The prior experience with a similar application in pediatric patients with congenital pulmonary valve stenosis was reported by Jean S. Kan earlier the same year [60, 61].

Field pioneers, like Albert P. Rocchini and Zuhdi Lababidi, acknowledged the findings of Pepine and Kan with percutaneous transluminal valvuloplasty as an effective and less invasive treatment than the open surgical valvulotomy [62, 63, 64].

Moving from the pulmonary valve to the AV, the first percutaneous transluminal retrograde balloon aortic valvuloplasty (BAV) was conducted by Lababidi on an 8-year-old patient with severe AVS in November 1982 [65, 66].

### 6.5 BAV in Treatment of Acquired Calcific AS 

The advancements mentioned above in transluminal balloon valvuloplasty led to the utilization of percutaneous BAV (formerly: “Percutaneous transluminal balloon catheter aortic valvuloplasty (PTAV)” in the original paper) in elderly patients with acquired severe calcific AVS by Alain Cribier in 1985 [67, 68, 69].

The short-term results were encouraging, with a trans-Aortic gradient reduction of more than 50% in all 3 cases and an immediate reduction in clinical symptoms.

Unfortunately, the high rate of recurrence of symptomatic AVS and the risk of significant aortic valve insufficiency limited the use of this technique. The pitfalls of BAV in that regard were indifferent to those of the surgical transaortic valvuloplasty (through digital or instrumental dilatation) described as early as 1958 by Bailey [70, 71, 72].

The accepted indications for BAV are nowadays limited to bringing hemodynamically unstable patients to SAVR or TAVI, temporarily treating patients in urgent need of noncardiac surgery, and palliating patients considered too sick for TAVI, but that are still in need of symptomatic relief [53].

### 6.6 The Era of Experimental, In-Animal Transcatheter Aortic Valve Implantation (TAVI)

**Fig. 3.**
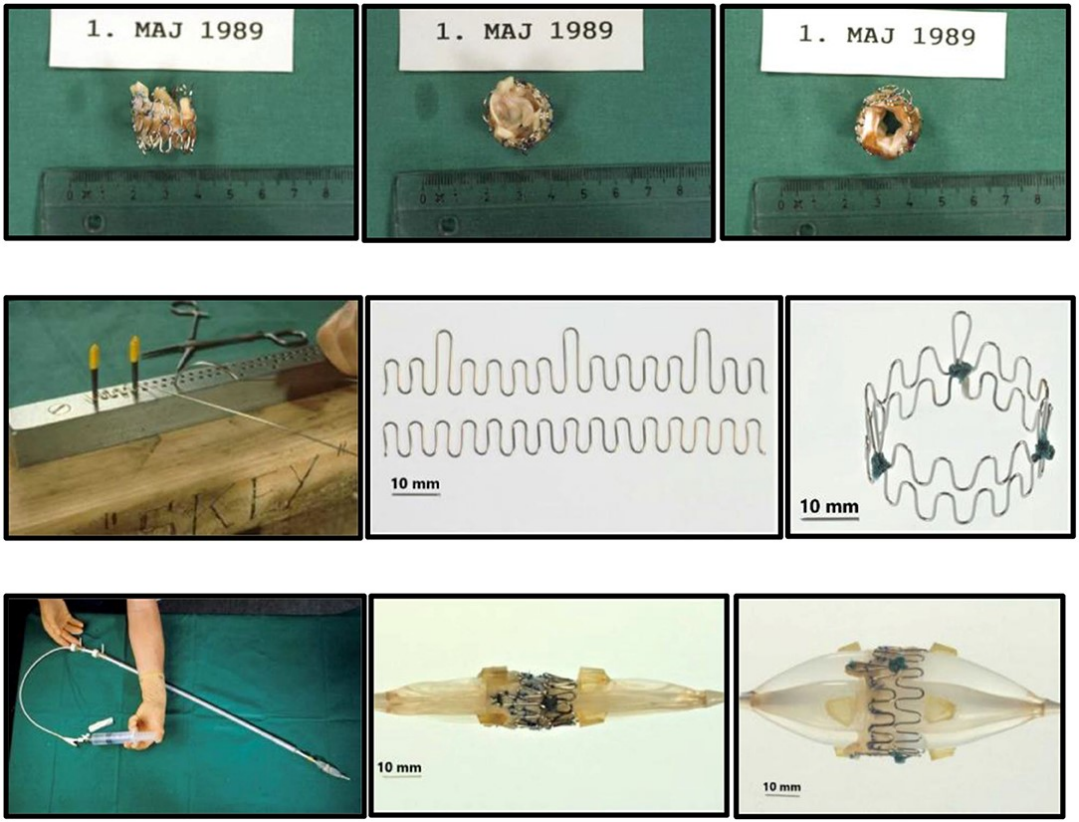
**“Prototype of TAVI valve and catheter technology. Top: The first-in-animal (FIA) valve implanted May 1. 1989. Middle: Later refinement of stent construction. Bottom: The 75 cm long, 41 Fr. introducer sheath with crimped and dilated TAVI valve on a three-foiled balloon aortic valvuloplasty dilatation catheter”**. (In Curtesy of Andersen HD [55]).

Henning Rud Andersen [58] designed and performed the FIA TAVI on an adult pig retrogradely with a self-made delivery system on May 1, 1989. The 75 cm long, 41 Fr. introducer sheath with a crimped and dilated TAVI valve on a three-foiled balloon aortic valvuloplasty dilatation catheter was conceptualized and built within 75 days after attending a lecture Palmaz gave during a conference in Scottsdale Arizona, USA in February 1989 [55]. The first metal stents were finger-folded using simple handheld tools from the hardware store. The biological valve material was an aortic allograft valve from another pig from the local slaughterhouse, hand-stitched on the stent (Fig. 3, [55]). As the 13.6 mm diameter sheath did not allow for percutaneous access, the abdominal aorta was prepared surgically. Despite these technical limitations, the first implantation was a success as a proof-of-concept [55, 56].

Dusan Pavcnik reported a percutaneous self-expandable mechanical valve that was successfully implanted in dogs shortly after the publication of the experience of Andersen *et al*. [58] in May 1992 [73].

Philippe Bonhoeffer also performed a preclinical evaluation with balloon implantation in the pulmonary artery in a lamb model [74], which eventually led to the first-in-human percutaneous balloon implantation in a 12-year-old boy with stenosis and insufficiency of a prosthetic valved conduit (from the right ventricle to the pulmonary artery) in 1990 [75].

Alain Cribier [76] performed and reported his first experience with balloon implanted valves in sheep in 2001 before getting the ethics committee’s approval for the first human TAVI in 2002 [22].

### 6.7 Percutaneous Transcatheter Implantation of an Aortic Valve Prosthesis for Calcific Aortic Stenosis 

The first percutaneous transcatheter implantation of an aortic valve prosthesis made of equine pericardium mounted on a stainless-steel balloon-expandable stent happened in a 57-year-old man with severe calcific AVS, cardiogenic shock, subacute leg ischemia, and other noncardiac comorbidities. The procedure was performed as an ultima ratio treatment, as the patient had declined again SAVR instead of surgical AVR, and a balloon valvuloplasty had already been performed with non-sustained results [22].

Cribier used both antegrade and retrograde approaches and achieved exceptional success considering the patients’ high frailty and comorbidities and the technique’s sophistication as reported in 2004 (I-REVIVE trial) [77]. The implantations were performed using mild sedation, without rapid right ventricular pacing or extracorporeal circulation. These early trials demonstrated above 75% procedural success with lasting hemodynamic performance at follow-up [78]. The main limitation observed was the incidence of 25% moderate—to severe paravalvular regurgitation, which occurred due to the availability of only a single size 23 mm valve prosthesis from Percutaneous Valve Technology (PVT, co-founded by Cribier) [78].

### 6.8 The Endorsement of TAVI by the Biomedical Industry and Its Rapid Development

The acquisition of PVT in January 2004 by Edwards Lifesciences (Irvine, CA, USA), a leading producer of surgical valve prostheses, triggered the rapid technological advancement of the prosthesis and the procedure.

The developments in the material technology allowed for miniaturization of the introducer systems so that, as envisioned by Anderson back in 1989, the retrograde approach was widely adopted with the advent of the transfemoral procedure in 2005. John G. Webb [79, 80] refined the retrograde technique in cooperation with Edwards and performed the implantation of the 23 mm and 26 mm Edwards SAPIEN valve (initially the Cribier-Edwards valve) through femoral access over a 22-/24-F pusher sheath with a deflectable Retroflex catheter.

Like its predecessor, the new Edwards-SAPIEN consisted of a tri-leaflet valve mounted on a balloon-expandable stainless-steel stent. However, the leaflets of this advanced model were constructed of pre-treated bovine pericardium instead of the equine pericardium to decrease the calcification rate. The size of the inner skirt was increased, and a 26 mm diameter valve was developed together with the 23 mm one to reduce the rate of perivalvular aortic insufficiency. The extended sheath allowed delivery directly into the descending aorta from the femoral artery. The retroflex catheter enhanced the atraumatic passage of the catheter and the mounted valve across the aortic arch.

Despite the slightly reduced sheath diameter of 22-F for the 23 mm valve, the incidence of small femoral access and the high degree of vessels’ calcification and tortuosity required further improvements in equipment and technique for a wider application of a transfemoral TAVI (TF-TAVI) [79, 80].

### 6.9 Other Transcatheter Approaches

Although we will mainly focus on the results of the TF-TAVI in the present review, few words should be spent on other alternatives, developed, and attempted since the early introduction of TAVI in the clinical practice.

John Webb [81] had performed the FIA transapical transcatheter aortic valve implantation (TA-TAVI) with an experimental self-expanding prosthesis in Vancouver in the year 2000. In cooperation with Edwards, his team further focused on the transfemoral retrograde approach, while other groups focused their efforts on the transapical approach [82].

The first human transapical TAVI was performed off-pump through a median sternotomy in Leipzig in 2006. The Vancouver group performed the first successful implant using a left anterior thoracotomy (intercostal access) shortly after [83]. Although the first implantations were performed through a reoriented Retroflex catheter, the purpose specific Ascendra delivery catheter became the standard transapical delivery system soon after.

Despite the common trend of superior overall procedural safety and faster recovery after TF-TAVI compared with TA-TAVI in numerous registries, the complementary role of TA-TAVI remains for high-risk patients that lack suitable femoral access [84, 85, 86, 87, 88].

A transaortic TAVI through a right anterolateral thoracotomy or a partial median sternotomy has also been proposed [86].

The trans-carotid, trans-subclavian, and trans-axillary approaches have also been proposed as alternative access routes [89, 90, 91, 92]. More recent results with these approaches will be discussed later in this review. Finally, other “unorthodox” access routes, like the transcutaneous apical and the trans-caval access to the abdominal aorta, have been used just in small series [93, 94, 95].

### 6.10 Self-Expandable TAVI—Prosthesis

The development of another TAVI-prosthesis was being pursued by the CoreValve company, a startup founded in 2001 that eventually was acquired by the biomedical giant Medtronic in 2009.

The early percutaneous aortic valve prostheses consisted of a metal stent and attached xenograft leaflets made initially of equine- or bovine pericardium. The two types of stents used were the stainless-steel balloon-expendable stent (as in the example of the Cribier-Edwards) and the self-expanding nitinol stent (as with the first example of its kind—the Medtronic CoreValve).

Jacques Seguin developed this first self-expanding transcatheter aortic valve prosthesis (CoreValve). The first human implantation of the prototype took place in India in 2002, and Eberhard Grube performed the first implantation in Europe in Siegburg, Germany, in 2005 [96]. The experience with this novel self-expanding valve prosthesis was published in 2006 as a registry study of 25 high-risk patients with severe AVS. These procedures were performed in general anesthesia with femoral extracorporeal bypass [97, 98]. The self-expandable prostheses were made of porcine pericardium (thinner and stiffer than bovine and equine pericardium) and allowed the transfemoral insertion of the delivery system through a smaller diameter sheath, initially 21-F and later18-F. This allowed the use of transfemoral access in a broader spectrum of patients and enabled also a subclavian access [99, 100, 101, 102]. The CoreValve stent was shaped so that the proximal diameter was slightly wider than the middle section of the stent. The presence of a prosthesis waist reduced the risk of ostial coronary occlusion through the fractured calcific native leaflet. The CoreValve was initially available in 2 sizes (26 mm and 29 mm). The armamentarium was later broadened with an additional 31 mm valve. The distal portion of the CoreValve nitinol stent was much broader compared to its proximal part. In this way, the valve frame could also achieve partial anchoring and stabilization within the supra-coronary/ascending aorta.

This modification also contributed to a better perpendicular alignment and self-centering of the axis of the stent to the annular plane. It was crucial as the first generation of the CoreValve was only marginally repositionable, and the angular control was minimal. Although this design offered a lower rate of coronary occlusion and annulus rupture, the need for a slightly infra-annular proximal landing for safe deployment resulted in a higher incidence of atrioventricular block. Regardless of these specific limitations, the indisputable success of this first-generation TAVI prosthesis in terms of clinical benefit is well documented [96, 97].

## 7. Clinical Evidence

### 7.1 Initial TAVI Studies and Registries

The feasibility of TAVI with the first-generation TAVI prostheses led to the Conformité Européenne (CE) approval in 2007. It allowed for the proliferation of TAVI in Europe. Germany, followed by France (and other European nations), was one of the early adopters of this new treatment. Several initial single- and multicenter studies and registries were thus implemented to observe and demonstrate the safety and efficacy of these new products, as summarized in Tables 3,4 [103].

**Table 3.** **Feasibility study results and early registries**.

Author/Register	Year	Access	Prostheses	Logistic Euro-score (%)	Device Success (%)*	30-Days-mortalitiy (%)	Stroke (%)	Study design	No. Cases
Cribier *et al*. (I-REVIVE, RECAST)	2006	TFa, TF	CE	27.0	75.0	22.2	3.7	SC [47]	36
Grube *et al*.	2005	TF	CV	11.0	84.0	20.0	12.0	SC [51]	25
Grube *et al*.	2007	TF	CV	23.4	88.0	12.0	10.0	MC [54]	86
Webb *et al*.	2007	TF	ES	28.0	86.0	12.0	4.0	SC [53]	50
Piazza *et al*.	2008	TF	CV	23.1	97.2	8.0	0.6	MC [71]	646
REVIVE II	2008	TF	CE, ES	29.9	88.0	13.2	***	MC [123]	105
REVIVAL II	2006	TF	CE, ES	34.1	87.3	7.3	9.2	MC [122]	55
Lichtenstein *et al*	2006	TA	CE	35.0	100	14.0	none	SC [60]	7
Walter *et al*	2007	TA	ES	27.1	96.7	10.0	none	SC [119]	30
Walter *et al*.	2007	TA	ES	26.8	93.2	13.6	3.4	MC [120]	59
Walter *et al*.	2008	TA	ES	27.6	100	8.0	none	[39]	50
Rodés-Cabau *et al*.	2008	TA/TF	ES	26.0	91.0	8.7	none	[118]	23
TRAVERCE	2008	TA	CE, ES	26.9	92.9	14.9	2.0	MC [121]	168
Svensson *et al*.	2008	TA	ES	35.5	90.0	17.5	none	MC [83]	40

I-REVIVE, Initial Registry of EndoVascular Implantation of Valves in Europe trial; RECAST, Registry of Endovascular Critical Aortic Stenosis Treatment trial; REVIVAL, PeRcutaneous EndoVascular Implantation of VALves trial; TRAVERCE, The initial multicenter feasibility trial for TA-AVI; SC, single center; MC, multicenter; TFa, transfemoral antegrade; TF, transfemoral retrograde; TA, transapical; ES, Edwards – SAPIEN; CE, Cribier – Edwards; CV, Corevalve; ***Unpublished data/TCT 2008.

**Table 4.** **Randomized trials SAVR vs. TAVI**.

Major randomized controlled trials TAVI vs. SAVR, patients with high perioperative risk		
Trial	PARTNER 1A	CoreValve High Risk
Valve prothesis	SAPIEN	CoreValve
Primary endpoint	All-cause death at 1 year	All-cause death at 1 year
Total patients randomized	699	795
Primary outcome	30 days: 3.4% vs. 6.5% (*p* = 0.07)	1 year: 14.2% vs. 19.1% (*p *< 0.001)
	1 year: 24.2% vs. 26.8% (*p* = 0.44)	
Major randomized controlled trials TAVI vs. SAVR, patients with intermediate perioperative risk		
Trial	PARTNER 2	SURTAVI
Valve prothesis	SAPIEN XT	CoreValve
Primary endpoint	All-cause death or disabling stroke at 2 years	All-cause death or disabling stroke at 2 years
Total patients randomized	2032	1660
Primary outcome	30 days: 6.1% vs. 8.0% (*p* = 0.11)	1 year: 12.6% vs. 14.0% (95% credible interval [Bayesian analysis] for difference, −5.2 to 2.3%; posterior probability of noninferiority, >0.999)
	2 years: 19.3% vs. 21.1%, *p* = 0.001 for non-inferiority, *p* = 0.33 for superiority	
Major randomized controlled trials, TAVI vs. SAVR, patients with low perioperative risk		
Trial	PARTNER 3	Evolut Low Risk
Valve prothesis	SAPIEN 3	CoreValve Evolut R
Primary endpoint	All-cause death, stroke or rehospitalization at 1 year	All-cause death or disabling stroke at 2 years
Total patients randomized	1000	1468
Primary outcome	1 year: 8.5% vs. 15.1%; absolute difference, −6.6 percentage points; 95% confidence interval [CI], −10.8 to −2.5; *p *< 0.001 for noninferiority	2 years: 5.3% vs. 6.7% (95% Bayesian credible interval for difference, −4.9 to 2.1; posterior probability of noninferiority, >0.999)
Major randomized controlled trials, TAVI vs. SAVR, “all-comers” and ongoing trials		
Trial	NOTION (all-comers)	NOTION 2 (low surgical risk)
Valve prothesis	CoreValve	Any CE-Mark approved transcatheter aortic bioprosthesis
Primary endpoint	All-cause death, disabling stroke or myocardial infarction at 1 year	All-cause death, stroke and myocardial infarction at 1 year
Total patients randomized	280	372 (estimated - clinicaltrials.gov/ct2/show/NCT02825134)
Primary outcome	1 year: 13.1% vs. 16.3%; (*p* = 0.43)	Ongoing

The **PARTNER EU** (Placement of Aortic Transcatheter Valve European Union) included 130 patients from 9 centers in Europe who underwent TAVI (transfemoral and transapical approach) with the Edwards SAPIEN valve between April 2007 and January 2008 (data presented at the EuroPCR meeting 2009). Thirty days and six months survival were 81.2 and 58.0% (TA) and 91.8 and 90.2% (TF).

The **SOURCE** (Edwards SAPIEN Aortic Bioprosthesis European Outcome) registry included 1123 high-risk patients who underwent TF- and TA-TAVR in 32 centers across Europe. Overall procedural success was 93.8% (significantly higher than earlier feasibility studies), with 30-day mortality rates of 6.3% and 10.3% for the TF- and TA approaches, respectively [104].

In the following years, many prospective randomized trials were designed and developed to document the safety and efficacy of TAVI versus SAVR in different patient populations.

Table 4 summarizes the landmarks that have supported TAVI introduction and popularization. From the introduction of TAVI and through the following years, trials have been designed to document TAVI applicability in cohorts of patients with decreasing operative risk (high-intermediate-and low operative risk).

The landmark **PARTNER Trials** (Placement of AoRTic TraNscathetER Valve Trial Edwards SAPIEN) were initiated in 2007 with the contribution of 26 centers. A total of 3015 high-risk SAVR candidates were screened, and 1057 patients were enrolled in the PARTNER 1 Trial. The primary endpoint of the PARTNER Trial was death from any cause at one year. The trial comprised two cohorts. Cohort A included 699 patients with high operative risk. Patients were randomized (1:1) to undergo either transfemoral (TF)-, transapical (TA)-TAVI (TF if suitable femoral access was available otherwise, TA), or SAVR [105].

The 358 remaining patients deemed inoperable, formed the Cohort B of the PARTNER 1 trial, and were randomized (1:1) to receive either TF-TAVI or standard therapy (which occasionally included BAV) [106].

In the Cohort B TF-TAVI demonstrated a significant superiority to optimal medical treatment (including BAV) regarding the following endpoints: all-Cause Mortality (30.7% vs. 50.7%; *p *< 0.001), cardiovascular mortality (19.6% vs. 41.9%; *p *< 0.001), re-hospitalization (22.3% vs. 44.1%; *p *< 0.001) and functional status. It should be noted that major vascular complications (16.2% vs. 1.1%; *p *< 0.001), major bleeding (22.3% vs. 11.2%; *p *< 0.001), as well as major strokes (5.0% vs. 1.1%; *p* = 0.06) occurred more frequently in the TAVI group [106].

In Cohort A, TF-/and TA-TAVI showed a noninferiority in the primary endpoints regarding survival. There was a statistically significantly higher incidence of major vascular complications at 30 days after TAVI compared with SAVR (11% vs. 3.2%; *p *< 0.001) and a higher incidence of major bleeding (19.5% vs. 9.3%; *p *< 0.001) and new-onset atrial fibrillation (16% vs. 8.6%; *p* = 0.006) after SAVR. From these results, TAVI arose as a viable alternative to SAVR in high-risk patients and became the new gold-standard therapy in patients with severe AVS unsuitable for SAVR. Upon the affirmative results of this trial, TAVI was approved by the Food and Drug Administration (FDA) for these indications in 2011 and 2012, respectively.

The **Medtronic CoreValve U.S. Pivotal** (high-risk) **Trial **(2010-2014-2019) was the first randomized controlled trial to demonstrate the superiority of TAVI vs. SAVR in high-risk AVS patients. A significantly lower all-cause 1-year mortality was documented in TAVI with the self-expandable Core Valve (TAVI 14.2% vs. SAVR 19.1%; *p *< 0.001). At 5-year follow-up, all-cause mortality rates were 55.3% for TAVI and 55.4% for SAVR. Subgroup analysis showed no differences in mortality. Cerebrovascular accident rates were 12.3% for TAVI and 13.2% for SAVR. Additionally, the study showed negligible severe prosthesis degeneration and intervention rates in both groups [107, 108].

### 7.2 Second & Third Generation TAVI Prostheses in Patients at Moderate and Low Surgical Risk

After the proof of concept of the TAVI procedure with the feasibility studies in inoperable patients and after the demonstration of TAVI noninferiority to the SAVR in high-risk patients (STS-Score >10%, or Log. Euro score >20%), it was now the time to refine the TAVI technology and expand the treatment to patients with lower surgical risk.

Generally speaking, for both balloon-expandable and self-expandable TAVI new-generation prostheses, the goal was to reduce invasiveness by minimizing the system’s sheath size, limit the risk of a paravalvular leak by optimizing valve frame sealing, preserve coronary access by slightly modifying valve’s frame design and geometry, and facilitate prosthesis implantation in order to achieve the best position and function.

The evolution of the prototypical balloon expendable TAVI prosthesis (Cribier-Edwards, 2002) into Edwards SAPIEN (2006), Edwards SAPIEN XT (2009), Edwards SAPIEN 3 (since 2012), and, lately, Edwards SAPIEN 3 Ultra (2019) is shown in Table 5.

**Table 5.** **Evolution of the balloon-expandable Edwards TAVI platform**.

	Cribier-Edwards	Edwards-Sapien	Sapien XT	Sapien 3
	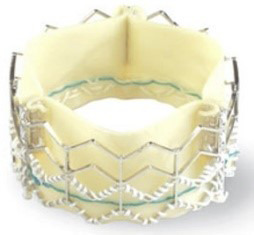	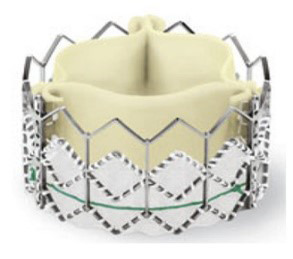	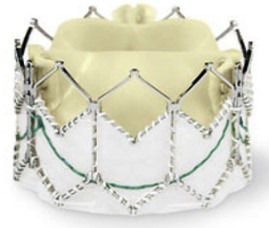	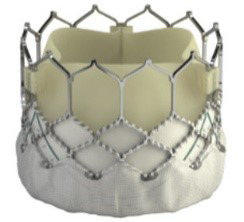
Available sizes (mm)	23	23/26	20, 23, 26/29	20, 23, 26/29
Introducer sheath size (Fr)	24	22/24	16/18	14/16
Valve material	Equine pericardium	Bovine pericardium	Bovine pericardium	Bovine pericardium
Internal pericardial wrap proportion	1/3	1/2	>1/2	>1/2
External pericardial wrap	No	No	No	Yes

The SAPIEN XT valve had two additional sizes and a lower profile. It could be crimped to a significantly lower diameter, allowing the delivery system to pass through a 16- to 18-F sheath. The delivery system diameter reduction continued and supported the development of an optimized Edwards SAPIEN 3 valve with an outer skirt to increase annular sealing. An expandable 14- to 16-F sheath enabled further reduction of femoral invasiveness, increasing the possibility of performing purely percutaneous femoral access.

Additionally, the most recent generations of balloon-expandable TAVI prostheses, the SAPIEN 3 and the SAPIEN 3 Ultra, have a taller stent frame with more giant cells in the upper row, facilitating the coronary ostia access. The newer model SAPIEN 3 Ultra has an even higher outer skirt made of synthetic material to facilitate healing and further improve the annular sealing.

The year 2012 marked the next generation of balloon expendable valves and the optimization of the prototypical self-expandable Medtronic CoreValve into the Evolute R prosthesis.

Table 6 summarizes the evolution of the Medtronic TAVI platform. Medtronic’s CoreValve system was the first-generation self-expandable valve that made it to the market. Large catheters (18-F to 24-F) were required for vascular access. The CoreValve Evolut R System was the second-generation TAVI device produced by Medtronic. The prosthesis had a shorter overall height with a preserved and extended pericardial skirt height for better annular sealing. It was resheathable and could be recaptured and repositioned during deployment to optimize final positioning. The entire system could be inserted without needing an additional access sheath, thereby reducing the profile of the delivery system down to 14-Fr.

**Table 6.** **Evolution of the self-expandable Medtronic TAVI platform**.

	CoreValve	Evolut R	Evolut PRO	Evolut PRO +
	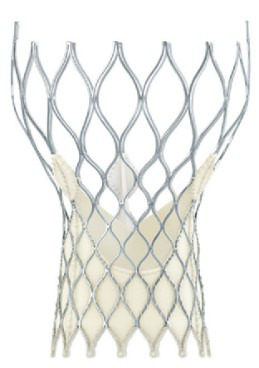	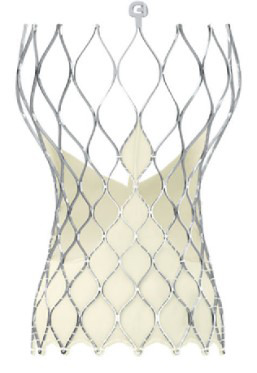	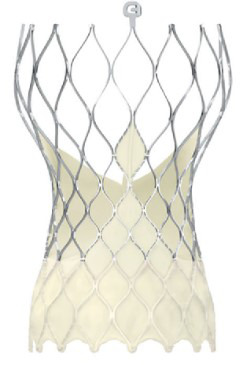	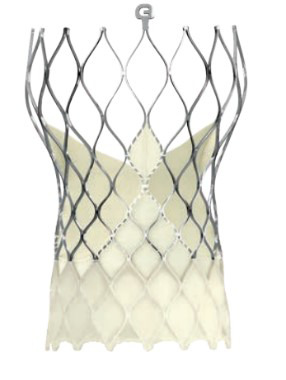
Available sizes (mm)	26, 29, 31	23, 26, 29/34	23, 26, 29	23, 26, 29/34
Minimum vessel diameter (mm)	6.0	5.0	5.5	5.0
Introducer sheath size (Fr)	18/20	14/16	16	14/18
Valve material	Porcine pericardium	Porcine pericardium	Porcine pericardium	Porcine pericardium
Complete recapturability	No	Yes	Yes	Yes
External pericardial wrap (external skirt)	No	No	No	Yes

The Evolut Pro and Evolut Pro + TAVI prostheses represent the most recent developments where an external pericardial wrap ensures further reduction in paravalvular leak occurrence, and an improved valve release system allows for a more precise valve positioning and final deployment. A reduced delivery catheter system size (14-F equivalent for the 23-26-29 mm valves and 18-F equivalent for the 34 mm valve) further decreases procedure invasiveness. The scientific evidence of the operative and clinical advantages achieved thanks to the device’s modifications are reported later in this review.

### 7.3 Additional TAVI Platforms

Although the Edwards and Medtronic TAVI platforms remain the most used ones, and the review and evaluation of additional TAVI prostheses exceed the intent of this manuscript, yet some noteworthy examples have offered unique advantages and challenges (Table 7).

**Table 7.** **Additional TAVI platforms**.

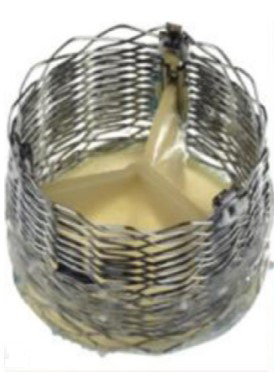	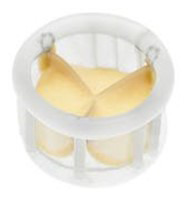	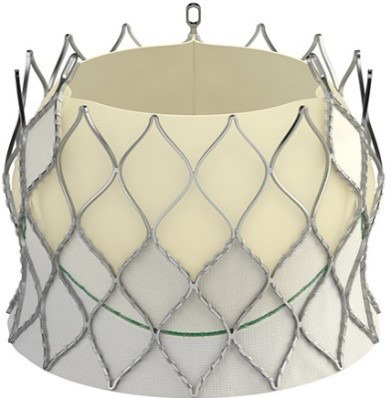
Lotus valve (Boston Scientific Inc., MN, USA)	Direct Flow valve (Direct Flow Medical Inc., CA, USA)	Centera Valve (Edwards Lifesciences Inc., CA, USA)
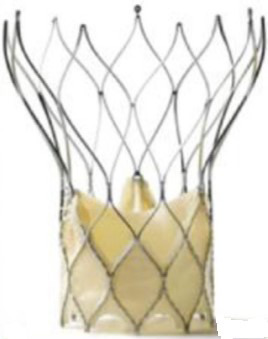	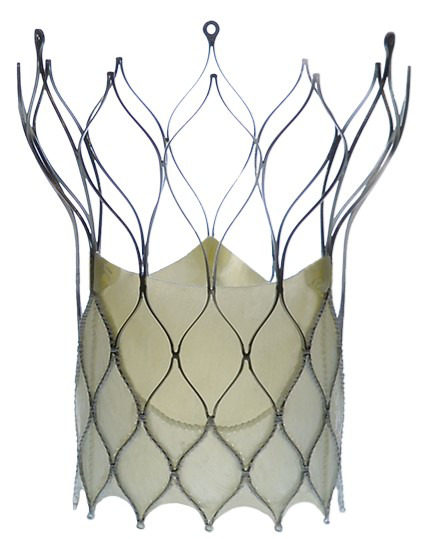	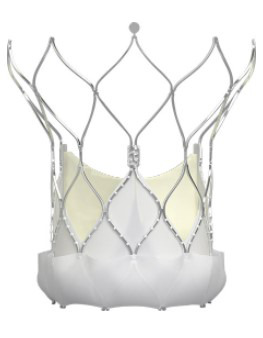
Portico valve (St. Jude Medical Inc., MN, USA)	BioValve (BIOTRONIK SE & Co. KG, Berlin, Germany)	Navitor (Abbott Laboratories Inc., Illinois, USA)
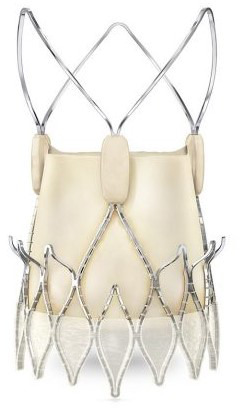	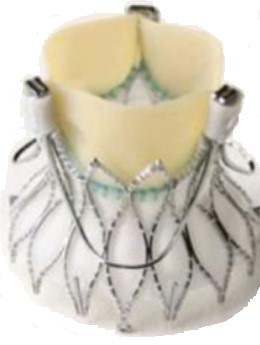	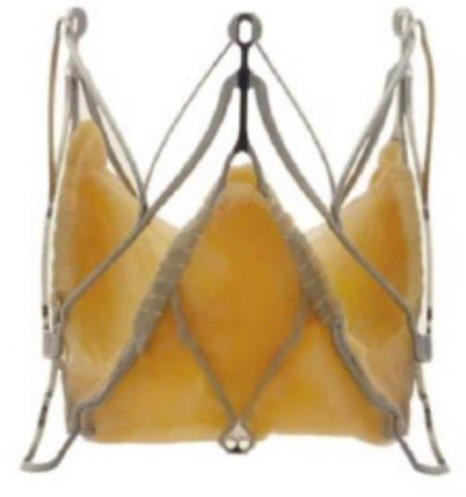
Acurate neo (since 2107 Boston Scientific Inc., MN, USA)	Engager valve (Medtronic Inc., MN, USA)	JenaClip valve (JenaValve Inc., Munich, Germany)

The Lotus™ valve (Boston Scientific, Marlborough, MA, USA) (introduction ~09/2013) was the first and only mechanically expandable TAVI prosthesis. It had a valve stent frame constructed of woven nitinol wires, which, when tensioned, caused the valve to decrease in height while increasing in diameter and rigidity. It allowed the system to be 100% repositionable and re-sheathable (before final release). This valve offered superior sealing with a higher radial force resulting in a negligible paravalvular leak rate. The elevated prosthesis radial force resulted in a high permanent pacemaker implantation rate, reaching up to 30% in some experiences. The Nordic Lotus-TAVR Registry and the REPRISE II trial (Repositionable Percutaneous Replacement of Stenotic Aortic Valve Through Implantation of Lotus Valve System–Randomized Clinical Evaluation), and the RESPOND post-marketing register showed consistent results [68, 109, 110]. The device went through several recalls between 2016–2020 due to difficulties with the release mechanism, and the Lotus Edge system was consequently discontinued.

Boston Scientific acquired the Symetis™ system (Ecublens, VD, Switzerland) in 2017. The system was subsequently improved and renamed (Accurate *neo*™). The Accurate self-expanding of the supra-annular valve has achieved favorable acute and longer-term results (as investigated in the SAVI-TF Post market Register) [111].

Some of the self-expandable TAVI prostheses like the Portico™ (St. Jude Medical Inc., MN, USA) and Accurate *neo*™ utilize long distal stent extensions to attain better supra coronary contact for superior annular alignment and additional fixation, just like the CoreValve device.

In some of the self-expandable TAVI prostheses, another structural innovation has been adding flexible arms extending above the native leaflets to facilitate better rotational orientation to the native commissures and coronary ostia. The Engager valve (Medtronic, Inc., Minneapolis, MN, USA), the Accurate *neo* 2 (Boston Scientific, Marlborough, MA, USA), and the JenaClip valve (JenaValve Inc., Munich, Germany) incorporate this feature in their original designs.

The Centera valve (Edwards Lifesciences, Irvine, CA, USA) was a self-expanding, nitinol-frame, bovine pericardial leaflet valve with a polyethylene terephthalate skirt available in 23- and 26-mm sizes. One of the main design choices was the lower height of the prostheses allowing self-centering and minimal ventricular protrusion [112]. This device was discontinued in 2019, and the leading manufacturer of balloon-expandable TAVI prostheses consolidated Edwards’s technical and marketing efforts with the Sapiens 3 Ultra series.

The Biovalve™ (Biotronik, Buelach, Switzerland) is another noteworthy self-expandable TAVI prosthesis. The first-in-man case with this valve was presented in 2015, and the BIOVALVE-1 and -2 feasibility trials were published recently. The valve’s performance was in line with other first-generation valves [113, 114].

The Direct Flow™ valve (Direct Flow Medical Inc., CA, USA)—consisted of a tubular fabric frame inflated with a rapidly setting polymerizing agent [115, 116, 117]. This CE-marked device showed auspicious performance, but its commercialization was discontinued in 2017 after failing to secure funding.

### 7.4 Broadening TAVI Indication Considering Major Trials and Technological Improvements

As of 2010, TAVI was indicated in the presence of high operative risk for SAVR [118]. At this point, the TAVI outcomes in intermediate-risk patients, presenting with a logistic EuroSCORE lower than 20% or a Society of Thoracic Surgeons (STS) score between 4% and 8% were already being evaluated in prospective randomized trials such as the **SURTAVI trial** (TF CoreValve™ vs. SAVR) and **PARTNER 2 trial** (Edwards SAPIEN-X vs. SAVR). Concurrently the SOURCE-XT, another multicenter registry including 2688 patients in 99 European centers, was investigating the performance of the second-generation Edwards—Sapien XT device. The results confirmed a marked decrease in vascular complications and bleeding and a decrease of one year all-cause mortality and cardiovascular mortality to 19.8% and 10.8%, respectively [119].

The PARTNER 2 trial enrolled 2032 intermediate-risk and 560 high-risk or inoperable patients from 57 centers to undergo either TF- or TA- TAVI (Edwards -SAPIEN or Edwards SAPIEN XT) or SAVR. Its primary endpoint was all-cause mortality or disabling stroke at two years.

The observations in the intermediate-risk cohort showed a noninferiority of TAVI with Edwards SAPIEN XT device (TF and TA) versus SAVR on rates of 30 days and two years mortality or disabling stroke. Furthermore, in the TF-TAVI group alone, there was a significantly lower rate of death or disabling stroke (TF-TAVI 16.8% vs. SAVR 20.4%, Hazard Ratio 0.79, 95% CI 0.62–1.00, *p* = 0.05).

TF-TAVI with the SAPIEN XT resulted in larger aortic-valve areas and lower rates of acute kidney injury, severe bleeding, and new-onset atrial fibrillation. In contrast, SAVR resulted in a lower incidence of major vascular complications and moderate to severe paravalvular aortic regurgitation [120]. These results led the FDA to extend approval of TAVI to intermediate-risk patients as well.

The SURTAVI trial’s primary endpoint was all-cause mortality or disabling stroke for TAVI vs. SAVR at two years.

Results, as published in 2017, reflected that TF-TAVI with CoreValve (in 84% of the cases) and Evolut R devices were a non-inferior alternative to SAVR (all-cause mortality or disabling stroke at two years TF-TAVI 12.6% vs. SAVR 14.0%, *p *< 0.05 for noninferiority) in patients with severe AS at intermediate surgical risk. The TAVI group was associated with higher rates of aortic regurgitation and permanent pacemaker implantation [121, 122].

The 2012 ESC/EACTS Guidelines stated that TAVI “should not be performed” in patients considered to have an intermediate risk (STS Score of 4%–8%) for surgery [123]. However, following the publication of those mentioned above, large-scale randomized controlled trials that concluded in favor of TAVI in the treatment of patients with an intermediate surgical risk, the European guidelines were modified in 2017 along with the AHA’s guidelines to categorize TAVI as a non-inferior and reasonable alternative to SAVR in these patients (class IIa recommendation) [124].

The progression and improvements in TAVI prostheses have been able to mitigate many of the complications and limitations of the first-generation TAVI prostheses. PARTNER II S3 refers to the nonrandomized cohorts of the PARTNER II Trial that were treated with the Sapien 3 valve. The 30-day mortality, major vascular complications, and stroke rate were the lowest reported in balloon-expandable TAVI trials. A rate of greater than mild paravalvular insufficiency of only 3.7% was observed at 30 days.

The PARTNER 3 trial started in 2016 with the “all-comers older than 65 years of age” principle [23]. The promising results with first- and second-generation TAVI prostheses in the high and intermediate surgical risk patient population in need of AVR facilitated the application of the therapy in elderly patients with lower surgical risk. A similar trial has been performed with the Medtronic Evolut R & Evolut PRO TAVI prostheses (Evolut Low-Risk Trial) [125].

Both trials are again randomized noninferiority trials in which TAVI is compared with SAVR in patients with severe AVS and low surgical risk (PARTNER 3 STS-score <4%, Evolut Low Risk ≤3%) with a primary endpoint of all-cause mortality, disabling stroke, and rehospitalization (PARTNER 3) at one (PARTNER 3) and two years (Evolut Low Risk).

In the PARTNER 3 trial, the primary outcome of all-cause mortality, stroke, or rehospitalization at one year, occurred in 8.5% of the TAVI group compared with 15.1% of the SAVR group (*p *< 0.001 for noninferiority, *p* = 0.001 for superiority). There was no statistically significant difference in the postprocedural permanent pacemaker-implantation rate in 1 year in the TAVI group vs. SAVR (7.3% vs. 5.4%, *p* = 0.21). The incidence of major bleeding (3.6% vs. 24.5%) and new-onset atrial fibrillation (5.0% vs. 39.5%) was significantly lower with TAVI compared to SAVR. The substantially lower post-operative hospital stays with TAVI and one-year rehospitalization rate of 1.4% with TAVI vs. 3.6% with SAVR (*p* = 0.029) emphasize even further the TAVI advantages.

In the Evolut Low-Risk trial, the second and third-generation Medtronic Evolut TAVI prostheses have demonstrated a noninferiority at two years in respect to the composite primary endpoint [125]. The primary endpoint of all-cause mortality or disabling stroke for TAVI vs. SAVR at 24 months was 5.3% vs. 6.7% (*p *< 0.05 for noninferiority, *p *> 0.05 for superiority).

The TAVI group had lower incidence of disabling stroke (0.5% vs. 1.7% at 30 days and 0.8% vs. 2.4% at 12 months), fewer bleeding complications (2.4% vs. 7.5% at 30 days; 3.2 % vs. 8.9% at 12 months), lower incidence of acute kidney injury (0.9% vs. 2.8% at 30 days and 12 months), lower atrial fibrillation occurrence (7.7% vs. 35.4% at 30 days; 9.8% vs. 38.3% at 12 months), and higher rate of permanent pacemaker implantation (17.4% vs. 6.1% at 30 days; 19.4% vs. 6.7% at 12 months). Moderate or severe total aortic regurgitation was present at 30 days in 3.5% of TAVI patients and 0.5% of SAVRs. The TAVI group consistently reported lower aortic-valve gradients (8.6 mmHg vs. 11.2 mmHg) and larger effective orifice areas (2.3 ${\mathrm{cm}}^{2}$ vs. 2.0 ${\mathrm{cm}}^{2}$). Severe patient–prosthesis mismatch occurred at 12 months in 1.8% of the patients in the TAVI group and 8.2% in the SAVRs [125].

The NOTION (Nordic Aortic Valve Intervention) was a smaller (280 patients) randomized control trial with an all-comers approach (with ~82% of participants at low risk for SAVR) that compared the outcomes of SAVR and TAVI with CoreValve™ [126, 127]. The study aimed to compare clinical outcomes and valve durability after eight years of follow-up. The results were mostly in line with that of the EVOLUTE Low-Risk trial, with higher rates of permanent pacemaker implantation and paravalvular leak in TAVI patients, higher rates of atrial fibrillation in the SAVR group, and no statistical difference in terms of all-cause death, stroke, and myocardial infarction [126, 127].

Finally, a recent meta-analysis of the present registries focused on all-cause mortality and stroke (follow-up length of two years) with TAVI vs. SAVR across the entire spectrum of surgical risk patients [128]. The analysis included over 12,000 patients and showed that TAVI was associated with a significant reduction of all-cause mortality compared to SAVR (HR 0.88; 95% CI 0.78–0.99; *p* = 0.030). The TAVI protective effect was consistent across the entire spectrum of surgical risk and irrespective of type of TAVI prostheses. Moreover, TAVI resulted in lower risk of strokes (HR 0.81; 95% CI 0.68–0.98; *p* = 0.028). SAVR had a lower risk of major vascular complications (HR 1.99; 95% CI 1.34–2.93; *p* = 0.001) and permanent pacemaker implantations (HR 2.27; 95% CI 1.47–3.64; *p *< 0.001) compared to TAVI.

### 7.5 Prostheses Durability

The superiority of third-generation TAVI prostheses in high, intermediate, and low-risk patients compared to SAVR at least at mid-term follow-up shows not only the potential of this treatment but also the necessity for further investigation of the long-term durability of the TAVI prostheses. Only in this way TAVI use in younger patients, with extended life expectancy, could be supported in the next future [129].

The five-year follow-up results of the PARTNER 2 trial confirm an exciting development regarding the durability that seems to improve from the early SAPIEN XT to the more recent SAPIEN 3 prostheses. Compared with SAVR prostheses, the SAPIEN XT had a higher 5-year rate of structural valve deterioration (SVD). In contrast, the third-generation SAPIEN 3 had an SVD rate that was not different from that observed in SAVR prostheses. In matched cohorts, SVD and SVD-related bioprosthetic valve failure (BFV) was significantly lower with SAPIEN 3 versus SAPIEN XT [130].

In the UK TAVI Trial (241 Patients treated with self-expandable and balloon-expandable TAVI prostheses between 2007 and 2011), 91% of the patients remained free of SVD between 5 and 10 years after TAVI, and with only one case developing severe SVD at 5.3 years [131].

In the NOTION trial, the 8-year estimated risk of SVD was lower after TAVI than after SAVR (13.9% vs. 28.3%; *p* = 0.0017), whereas the risk of bioprosthetic valve failure was similar (8.7% vs. 10.5%; *p* = 0.61) [127, 132].

A recent network metanalysis of 10 randomized trials was performed with 5-year follow-up data for echocardiographic outcomes and the most extended available follow-up data for clinical outcomes. Self-expandable TAVI valves demonstrated significantly larger effective orifice area, lower mean trans-valvular gradient, and less frequent PVD compared with balloon-expandable TAVI and SAVR prostheses [133].

The EAPCI registry and the STOP-AS RHU (Search Treatment and Improve Outcome of Patients with Aortic Stenosis, Recherche Hospital-Universitaire) French registry, and the planned 10-year follow-up of the ongoing low-risk and all-comers trials and the real-world registries will further clarify the long-term durability of TAVI bioprostheses.

### 7.6 TAVI for Bicuspid Aortic Valve

As previously elucidated, bicuspid AVS is nowadays routinely treated with TAVI. Although we have reached the *status quo* after collecting solid scientific evidence, patients with bicuspid AVS should be evaluated thoroughly to determine the exact anatomy of the landing zone and the consequent TAVI strategy. In a metanalysis comprising 189,693 patients, Zghouzi *et al*. [134] have shown no difference in TAVI for bicuspid vs. tricuspid AVS regarding all-cause mortality, cardiovascular mortality, myocardial infarction, vascular complications, acute kidney injury, coronary occlusion, annulus rupture, and reintervention/reoperation. The incidence of stroke, paravalvular leak, and the need for a pacemaker were less in the tricuspid AVS group [134].

A roadmap for TAVI in bicuspid AV should consider the different anatomical phenotypes, the evolving evidence, the patient-specific features, the tailored procedural planning, and the long-term follow-up results [135]. Future technical improvement of the prostheses will possibly continue to support the TAVI feasibility in bicuspid anatomy.

### 7.7 Alternative Vascular Approaches

Trans-carotid and trans-subclavian routes have been recently revisited for selected patients with prohibitive femoral-iliac access. In their metanalysis, Faroux *et al*. [136] have included over 70,000 patients for the evaluation of the impact of the TAVI arterial approach. After risk adjustment, a transcarotid/transsubclavian approach was not associated with an increased risk of 30-day death, bleeding, or vascular complication. The working group from the FRANCE-TAVI registry has proposed a pre-specified propensity score-based matching between patients undergoing TAVI via the femoral or alternative vascular approaches (carotid and subclavian artery mainly). Non-femoral TAVI was associated with similar outcomes, except for a 2-fold lower rate of major vascular complications and unplanned vascular repairs, compared to femoral TAVI [137].

## 8. Conclusions

In the present manuscript, we have taken the readers through the long and not always straightforward “travel” that has brought many colleagues and patients to achieve the *status quo* in severe AVS management. Thanks to the many actors involved, today, AVR in patients with AVS can be safely performed under local anesthesia, through a fully percutaneous approach, without using cardiopulmonary bypass and cardioplegic arrest. TAVI has revolutionized the way we treat AVS today. Its success and acceptance in the medical arena are witnessed by the fact that according to the most recent guidelines, patients 75 years or older or with a high risk for SAVR, defined as STS-PROM/EuroScore II >8%, and being in general suitable for a transfemoral TAVI procedure are suggested, after heart-team discussion, to undergo TAVI [6, 7].

If the excellent results of TAVI are confirmed in younger patients and patients with even lower surgical risk, TAVI will become the primary treatment for symptomatic severe AVS. The longer-term performance of TAVI tissue prostheses will also need to be investigated according to the extended life expectancy of future TAVI candidates.

When writing this review, newly designed TAVI prostheses are under evaluation and promise improved acute and long-term performance. The two major players in the TAVI market, i.e., Medtronic and Edwards, will soon launch the Evolut FX and SAPIEN 4 with added features for more precise implantation and orientation within the native aortic anatomy and leaflets anti-calcification treatment (SAPIEN 4).

Durability of the existing TAVI tissue prostheses will be challenged, and attention will also be focused on alternative materials for TAVI valve manufacturing. In the future, prosthetic valve tissue fabrication may lead to the customized development of polymeric non-animal derived TAVI prostheses that will mimic the native valves in structure, function, and mechanical properties and, for this reason, will have extended durability [138, 139].

## Abbreviations

ACC, American College of Cardiology; AHA, American Heart Association; AVA, Aortic Valve Area; AVAi, indexed Aortic Valve Area; AVS, Aortic valve stenosis; BP, blood pressure; EACTS, European Association for Cardio-Thoracic Surgery; ESC, European Society of Cardiology; LVEF, left ventricular ejection fraction; SAVR, surgical aortic valve replacement; SVi, indexed stroke volume; TAVI, transcatheter aortic valve implantation; BAV, Balloon aortic valvuloplasty; SAV, surgical aortic valvulotomy; PPM, Patient-Prosthesis-Mismatch.

## References

[1] Iung B, Delgado V, Rosenhek R, Price S, Prendergast B, Wendler O (2019). Contemporary Presentation and Management of Valvular Heart Disease: The EURObservational Research Programme Valvular Heart Disease II Survey. *Circulation*.

[2] Eveborn GW, Schirmer H, Heggelund G, Lunde P, Rasmussen K (2013). The evolving epidemiology of valvular aortic stenosis. The Tromsø Study. *Heart*.

[3] Osnabrugge RL, Mylotte D, Head SJ, Van Mieghem NM, Nkomo VT, LeReun CM (2013). Aortic stenosis in the elderly: disease prevalence and number of candidates for transcatheter aortic valve replacement: a meta-analysis and modeling study. *Journal of the American College of Cardiology*.

[4] Joseph J, Naqvi SY, Giri J, Goldberg S (2017). Aortic Stenosis: Pathophysiology, Diagnosis, and Therapy. *The American Journal of Medicine*.

[5] Ancona R (2020). Epidemiology of aortic valve stenosis (AS) and of aortic valve incompetence (AI): is the prevalence of AS/AI similar in different parts of the world. *Journal of Cardiology Practice*.

[6] Otto CM, Nishimura RA, Bonow RO, Carabello BA, Erwin JP, Gentile F (2021). 2020 ACC/AHA Guideline for the Management of Patients With Valvular Heart Disease: A Report of the American College of Cardiology/American Heart Association Joint Committee on Clinical Practice Guidelines. *Circulation*.

[7] Vahanian A, Beyersdorf F, Praz F, Milojevic M, Baldus S, Bauersachs J (2022). ESC/EACTS Scientific Document Group. 2021 ESC/EACTS Guidelines for the management of valvular heart disease. *European Heart Journal*.

[8] Dweck MR, Boon NA, Newby DE (2012). Calcific Aortic Stenosis. *Journal of the American College of Cardiology*.

[9] Carabello BA, Paulus WJ (2009). Aortic stenosis. *The Lancet*.

[10] Bates ER (2011). Treatment Options in Severe Aortic Stenosis. *Circulation*.

[11] Emanuel R, Withers R, O’Brien K, Ross P, Feizi O (1978). Congenitally bicuspid aortic valves. Clinicogenetic study of 41 families. *Heart*.

[12] Fedak PWM, Verma S, David TE, Leask RL, Weisel RD, Butany J (2002). Clinical and Pathophysiological Implications of a Bicuspid Aortic Valve. *Circulation*.

[13] Shah SY, Higgins A, Desai MY (2018). Bicuspid aortic valve: Basics and beyond. *Cleveland Clinic Journal of Medicine*.

[14] Soto B, Bargeron LM, Diethelm E (1985). Ventricular septal defect. *Seminars in Roentgenology*.

[15] Siu SC, Silversides CK (2010). Bicuspid Aortic Valve Disease. *Journal of the American College of Cardiology*.

[16] Vincent F, Ternacle J, Denimal T, Shen M, Redfors B, Delhaye C (2021). Transcatheter Aortic Valve Replacement in Bicuspid Aortic Valve Stenosis. *Circulation*.

[17] Kumar RK (2013). Rheumatic fever & rheumatic heart disease: the last 50 years. *The Indian Journal of Medical Research*.

[18] Guilherme L (2005). Rheumatic fever: how S. pyogenes-primed peripheral T cells trigger heart valve lesions. *Annals of the New York Academy of Sciences*.

[19] Chopra P (2007). Pathology and pathogenesis of rheumatic heart disease. *Indian Journal of Pathology & Microbiology*.

[20] Dangas G, Khan S, Curry BH, Kini AS, Sharma SK (1999). Angina pectoris in Severe Aortic Stenosis. *Cardiology*.

[21] Szerlip M, Arsalan M, Mack MC, Filardo G, Worley C, Kim RJ (2017). Usefulness of Balloon Aortic Valvuloplasty in the Management of Patients with Aortic Stenosis. *The American Journal of Cardiology*.

[22] Cribier A, Eltchaninoff H, Bash A, Borenstein N, Tron C, Bauer F (2002). Percutaneous Transcatheter Implantation of an Aortic Valve Prosthesis for Calcific Aortic Stenosis. *Circulation*.

[23] Mack MJ, Leon MB, Thourani VH, Makkar R, Kodali SK, Russo M (2019). Transcatheter Aortic-Valve Replacement with a Balloon-Expandable Valve in Low-Risk Patients. *The New England Journal of Medicine*.

[24] Leon MB, Mack MJ, Hahn RT, Thourani VH, Makkar R, Kodali SK (2021). Outcomes 2 Years After Transcatheter Aortic Valve Replacement in Patients at Low Surgical Risk. *Journal of the American College of Cardiology*.

[25] Hufnagel CA, Harvey WP, Rabil PJ, McDermott TF (1954). Surgical Correction of Aortic Insufficiency. *Surgery*.

[26] Harken DE, Soroff HS, Taylor WJ, Lefemine AA, Gupta SK, Lunzer S (1960). Partial and complete prostheses in aortic insufficiency. *The Journal of Thoracic and Cardiovascular Surgery*.

[27] Harken DE, Black H, Taylor WJ, Thrower WB, Soroff HS (1958). The surgical correction of calcific aortic stenosis in adults; Results in The First 100 Consecutive Transaortic Valvuloplasties. *Journal of Thoracic Surgery*.

[28] Lillehei CW, Gott VL, DeWall RA, Varco RL (1958). The surgical treatment of stenotic or regurgitant lesions of the mitral and aortic valves by direct vision utilizing a pump-oxygenator. *The Journal of Thoracic Surgery*.

[29] Starr A, Edwards ML (1961). Mitral Replacement: clinical experience with a ball-valve prosthesis. *Annals of Surgery*.

[30] Schoen FJ, Morse D, Steiner RM, Fernandez J (1985). Pathology of cardiac valve replacement. *Guide to prosthetic cardiac valves*.

[31] Yoganathan AP, Corcoran WH, Harrison EC, Carl JR (1978). The Björk-Shiley aortic prosthesis: flow characteristics, thrombus formation and tissue overgrowth. *Circulation*.

[32] Björk VO (1969). A New Tilting Disc Valve Prosthesis. *Scandinavian Journal of Thoracic and Cardiovascular Surgery*.

[33] Forman R, Gersh BJ, Fraser R, Beck W (1978). Hemodynamic assessment of Lillehei-Kaster tilting disc aortic and mitral prostheses. *The Journal of Thoracic and Cardiovascular Surgery*.

[34] Kalke BR, Carlson RG, Lillehei CW (1968). Hingeless double-leaflet prosthetic heart valve for aortic, mitral or tricuspid position. *Biomedical sciences instrumentation*.

[35] Kleine P, Hasenkam MJ, Nygaard H, Perthel M, Wesemeyer D, Laas J (2000). Tilting disc versus bileaflet aortic valve substitutes: intraoperative and postoperative hemodynamic performance in humans. *The Journal of Heart Valve Disease*.

[36] Torella M, Torella D, Chiodini P, Franciulli M, Romano G, De Santo L (2010). LOWERing the INtensity of oral anticoaGulant Therapy in patients with bileaflet mechanical aortic valve replacement: Results from the “LOWERING-IT” Trial. *American Heart Journal*.

[37] Schlitt A, von Bardeleben RS, Ehrlich A, Eimermacher A, Peetz D, Dahm M (2003). Clopidogrel and aspirin in the prevention of thromboembolic complications after mechanical aortic valve replacement (CAPTA). *Thrombosis Research*.

[38] Koertke H, Zittermann A, Tenderich G, Wagner O, El-Arousy M, Krian A (2007). Low-dose oral anticoagulation in patients with mechanical heart valve prostheses: final report from the early self-management anticoagulation trial II. *European Heart Journal*.

[39] Puskas J, Gerdisch M, Nichols D, Quinn R, Anderson C, Rhenman B (2014). Reduced anticoagulation after mechanical aortic valve replacement: Interim results from the Prospective Randomized on-X Valve Anticoagulation Clinical Trial randomized Food and Drug Administration investigational device exemption trial. *The Journal of Thoracic and Cardiovascular Surgery*.

[40] Ross DN (1962). Homograft replacement of the aortic valve. *The Lancet*.

[41] Ross DN (1995). Evolution of the homograft valve. *The Annals of Thoracic Surgery*.

[42] Carpentier A, Lemaigre G, Robert L, Carpentier S, Dubost C, Gerbode F (1969). Biological factors affecting long-term results of valvular heterografts. *The Journal of Thoracic and Cardiovascular Surgery*.

[43] Ionescu MI (2014). *The begining. The pericardial heart valve*.

[44] Rahimtoola SH (1978). The problem of valve prosthesis-patient mismatch. *Circulation*.

[45] Desai ND, Merin O, Cohen GN, Herman J, Mobilos S, Sever JY (2004). Long-Term Results of Aortic Valve Replacement with the St. Jude Toronto Stentless Porcine Valve. *The Annals of Thoracic Surgery*.

[46] Manouguian S, Seybold-Epting W (1979). Patch enlargement of the aortic valve ring by extending the aortic incision into the anterior mitral leaflet. New operative technique. *The Journal of Thoracic and Cardiovascular Surgery*.

[47] Coutinho GF, Correia PM, Paupério G, de Oliveira F, Antunes MJ (2011). Aortic root enlargement does not increase the surgical risk and short-term patient outcome. *European Journal of Cardio-Thoracic Surgery*.

[48] Russo M, Taramasso M, Guidotti A, Pozzolli A, Nietilspach F, von Segesser L (2017). The evolution of surgical valves. *Cardiovascular Medicine*.

[49] Martínez-Comendador J, Castaño M, Gualis J, Martín E, Maiorano P, Otero J (2017). Sutureless aortic bioprosthesis. *Interactive CardioVascular and Thoracic Surgery*.

[50] Shrestha M, Fischlein T, Meuris B, Flameng W, Carrel T, Madonna F (2016). European multicentre experience with the sutureless Perceval valve: clinical and haemodynamic outcomes up to 5 years in over 700 patients. *European Journal of Cardio-Thoracic Surgery*.

[51] Al-Sarraf N, Thalib L, Hughes A, Houlihan M, Tolan M, Young V (2011). Cross-clamp time is an independent predictor of mortality and morbidity in low- and high-risk cardiac patients. *International Journal of Surgery*.

[52] Williams ML, Flynn CD, Mamo AA, Tian DH, Kappert U, Wilbring M (2020). Long-term outcomes of sutureless and rapid-deployment aortic valve replacement: a systematic review and meta-analysis. *Annals of Cardiothoracic Surgery*.

[53] Attia RQ, Raja SG (2021). Surgical pericardial heart valves: 50 Years of evolution. *International Journal of Surgery*.

[54] Forscher BK (1963). Chaos in the Brickyard. *Science*.

[55] Andersen HR (2021). How Transcatheter Aortic Valve Implantation (TAVI) Was Born: The Struggle for a New Invention. *Frontiers in Cardiovascular Medicine*.

[56] Dotter CT, Judkins MP (1964). Transluminal Treatment of Arteriosclerotic Obstruction. Description of a new technique and a preliminary report of its application. *Circulation*.

[57] Grüntzig AR, Senning A, Siegenthaler WE (1979). Nonoperative Dilatation of Coronary-Artery Stenosis: percutaneous transluminal coronary angioplasty. *New England Journal of Medicine*.

[58] Andersen HR, Knudsen LL, Hasenkam JM (1992). Transluminal implantation of artificial heart valves. Description of a new expandable aortic valve and initial results with implantation by catheter technique in closed chest pigs. *European Heart Journal*.

[59] Pepine CJ, Gessner IH, Feldman RL (1982). Percutaneous balloon valvuloplasty for pulmonic valve stenosis in the adult. *The American Journal of Cardiology*.

[60] Kan JS, White RI, Mitchell SE, Anderson JH, Gardner TJ (1984). Percutaneous transluminal balloon valvuloplasty for pulmonary valve stenosis. *Circulation*.

[61] Kan JS, White RI, Mitchell SE, Gardner TJ (1982). Percutaneous Balloon Valvuloplasty: a New Method for Treating Congenital Pulmonary-Valve Stenosis. *New England Journal of Medicine*.

[62] Lababidi Z, Wu J (1983). Percutaneous balloon pulmonary valvuloplasty. *The American Journal of Cardiology*.

[63] Rocchini AP, Kveselis DA, Crowley D, Dick M, Rosenthal A (1984). Percutaneous balloon valvuloplasty for treatment of congenital pulmonary valvular stenosis in children. *Journal of the American College of Cardiology*.

[64] Walls JT, Lababidi Z, Curtis JJ, Silver D (1984). Assessment of percutaneous balloon pulmonary and aortic valvuloplasty. *The Journal of Thoracic and Cardiovascular Surgery*.

[65] Lababidi Z, Wu J, Walls JT (1984). Percutaneous balloon aortic valvuloplasty: Results in 23 patients. *The American Journal of Cardiology*.

[66] Lababidi Z (1983). Aortic balloon valvuloplasty. *American Heart Journal*.

[67] Cribier A, Savin T, Berland J, Rocha P, Mechmeche R, Saoudi N (1987). Percutaneous transluminal balloon valvuloplasty of adult aortic stenosis: Report of 92 cases. *Journal of the American College of Cardiology*.

[68] Cribier A, Saoudi N, Berland J, Savin T, Rocha P, Letac B (1986). Percutaneous transluminal valvuloplasty of acquired aortic stenosis in elderly patients: an alternative to valve replacement. *The Lancet*.

[69] Cribier A, Remadi F, Koning R, Rath P, Stix G, Letac B (1992). Emergency balloon valvuloplasty as initial treatment of patients with aortic stenosis and cardiogenic shock. *The New England Journal of Medicine*.

[70] Hill GD, Ginde S, Rios R, Frommelt PC, Hill KD (2016). Surgical Valvotomy Versus Balloon Valvuloplasty for Congenital Aortic Valve Stenosis: a Systematic Review and Meta‐Analysis. *Journal of the American Heart Association*.

[71] Hostetler MD, Dunn MI (1992). Percutaneous balloon aortic valvuloplasty: Dr. Bailey Revisited. *Journal of the American College of Cardiology*.

[72] Walther T, Falk V, Kempfert J, Borger MA, Fassl J, Chu MWA (2008). Transapical minimally invasive aortic valve implantation; the initial 50 patients. *European Journal of Cardio-Thoracic Surgery*.

[73] Pavcnik D, Wright KC, Wallace S (1992). Development and initial experimental evaluation of a prosthetic aortic valve for transcatheter placement. Work in progress. *Radiology*.

[74] Bonhoeffer P, Boudjemline Y, Saliba Z, Hausse AO, Aggoun Y, Bonnet D (2000). Transcatheter Implantation of a Bovine Valve in Pulmonary Position: a lamb study. *Circulation*.

[75] Bonhoeffer P, Boudjemline Y, Saliba Z, Merckx J, Aggoun Y, Bonnet D (2000). Percutaneous replacement of pulmonary valve in a right-ventricle to pulmonary-artery prosthetic conduit with valve dysfunction. *The Lancet*.

[76] Cribier A, Eltchaninoff H, Bareinstein N, Daniel P, Laborde F, Leon MB (2001). Trans-catheter implantation of balloon-expandable prosthetic heart valves: early results in an animal model. *Circulation*.

[77] Cribier A, Eltchaninoff H, Tron C, Bauer F, Agatiello C, Sebagh L (2004). Early experience with percutaneous transcatheter implantation of heart valve prosthesis for the treatment of end-stage inoperable patients with calcific aortic stenosis. *Journal of the American College of Cardiology*.

[78] Cribier A, Eltchaninoff H, Tron C, Bauer F, Agatiello C, Nercolini D (2006). Treatment of calcific aortic stenosis with the percutaneous heart valve: mid-term follow-up from the initial feasibility studies: the French experience. *Journal of the American College of Cardiology*.

[79] Webb JG, Chandavimol M, Thompson CR, Ricci DR, Carere RG, Munt BI (2006). Percutaneous Aortic Valve Implantation Retrograde from the Femoral Artery. *Circulation*.

[80] Webb JG, Pasupati S, Humphries K, Thompson C, Altwegg L, Moss R (2007). Percutaneous Transarterial Aortic Valve Replacement in Selected High-Risk Patients with Aortic Stenosis. *Circulation*.

[81] Webb JG, Binder RK (2012). Transcatheter aortic valve implantation: the evolution of prostheses, delivery systems and approaches. *Archives of Cardiovascular Diseases*.

[82] Walther T, Falk V, Borger MA, Dewey T, Wimmer-Greinecker G, Schuler G (2007). Minimally invasive transapical beating heart aortic valve implantation—proof of concept. *European Journal of Cardio-Thoracic Surgery*.

[83] Ye J, Cheung A, Lichtenstein SV, Carere RG, Thompson CR, Pasupati S (2006). Transapical aortic valve implantation in humans. *The Journal of Thoracic and Cardiovascular Surgery*.

[84] Svensson LG, Dewey T, Kapadia S, Roselli EE, Stewart A, Williams M (2008). United States feasibility study of transcatheter insertion of a stented aortic valve by the left ventricular apex. *The Annals of Thoracic Surgery*.

[85] Rodés-Cabau J, Dumont E, De LaRochellière R, Doyle D, Lemieux J, Bergeron S (2008). Feasibility and Initial Results of Percutaneous Aortic Valve Implantation Including Selection of the Transfemoral or Transapical Approach in Patients with Severe Aortic Stenosis. *The American Journal of Cardiology*.

[86] Blackstone EH, Suri RM, Rajeswaran J, Babaliaros V, Douglas PS, Fearon WF (2015). Propensity-matched comparisons of clinical outcomes after transapical or transfemoral transcatheter aortic valve replacement: a placement of aortic transcatheter valves (PARTNER)-I trial substudy. *Circulation*.

[87] Biancari F, Rosato S, D’Errigo P, Ranucci M, Onorati F, Barbanti M (2016). Immediate and Intermediate Outcome After Transapical Versus Transfemoral Transcatheter Aortic Valve Replacement. *The American Journal of Cardiology*.

[88] Overtchouk P, Modine T (2018). Alternate Access for TAVI: Stay Clear of the Chest. *Interventional Cardiology Review*.

[89] Sacha J, Krawczyk K, Gwóźdź W, Bugajski J, Perkowski T, Hobot J (2020). Fully Percutaneous Transaxillary Aortic Valve Replacement with Effective Bailout Plan for Vascular Complications. *JACC: Cardiovascular Interventions*.

[90] Oteo JF, Trillo R, García-Touchard A, Fernández-Díaz JA, Cavero MA, Goicolea J (2013). A first Case of Totally Percutaneous Transaxillary Aortic Valve Implantation. *Revista EspañOla De Cardiología*.

[91] Azmoun A, Amabile N, Ramadan R, Ghostine S, Caussin C, Fradi S (2014). Transcatheter aortic valve implantation through carotic artery access under local anaesthesia. *European Journal of Cardio-Thoracic Surgery*.

[92] Kirker EB, Hodson RW, Spinelli KJ, Korngold EC (2017). The Carotid Artery as a Preferred Alternative Access Route for Transcatheter Aortic Valve Replacement. *The Annals of Thoracic Surgery*.

[93] Greenbaum AB, O’Neill WW, Paone G, Guerrero ME, Wyman JF, Cooper RL (2014). Caval-Aortic Access to Allow Transcatheter Aortic Valve Replacement in otherwise Ineligible Patients: initial human experience. *Journal of the American College of Cardiology*.

[94] Halabi M, Ratnayaka K, Faranesh AZ, Chen MY, Schenke WH, Lederman RJ (2013). Aortic Access from the Vena Cava for Large Caliber Transcatheter Cardiovascular Interventions: pre-clinical validation. *Journal of the American College of Cardiology*.

[95] Greenbaum AB, Babaliaros VC, Chen MY, Stine AM, Rogers T, O’Neill WW (2017). Transcaval Access and Closure for Transcatheter Aortic Valve Replacement: A Prospective Investigation. *Journal of the American College of Cardiology*.

[96] Grube E, Laborde JC, Zickmann B, Gerckens U, Felderhoff T, Sauren B (2005). First report on a human percutaneous transluminal implantation of a self-expanding valve prosthesis for interventional treatment of aortic valve stenosis. *Catheterization and Cardiovascular Interventions*.

[97] Grube E, Laborde JC, Gerckens U, Felderhoff T, Sauren B, Buellesfeld L (2006). Percutaneous Implantation of the CoreValve Self-Expanding Valve Prosthesis in High-Risk Patients with Aortic Valve Disease: the Siegburg first-in-man study. *Circulation*.

[98] Grube E, Schuler G, Buellesfeld L, Gerckens U, Linke A, Wenaweser P (2007). Percutaneous aortic valve replacement for severe aortic stenosis in high-risk patients using the second- and current third-generation self-expanding CoreValve prosthesis: device success and 30-day clinical outcome. *Journal of the American College of Cardiology*.

[99] Kumar R, Latib A, Colombo A, Ruiz CE (2014). Self-Expanding Prostheses for Transcatheter Aortic Valve Replacement. *Progress in Cardiovascular Diseases*.

[100] Ruge H, Lange R, Bleiziffer S, Hutter A, Mazzitelli D, Will A (2008). First successful aortic valve implantation with the CoreValve ReValving System via right subclavian artery access: a case report. *The Heart Surgery Forum*.

[101] Asgar AW, Mullen MJ, Delahunty N, Davies SW, Dalby M, Petrou M (2009). Transcatheter aortic valve intervention through the axillary artery for the treatment of severe aortic stenosis. *The Journal of Thoracic and Cardiovascular Surgery*.

[102] Fraccaro C, Napodano M, Tarantini G, Gasparetto V, Gerosa G, Bianco R (2009). Expanding the eligibility for transcatheter aortic valve implantation the trans-subclavian retrograde approach using: the III generation CoreValve revalving system. *JACC: Cardiovascular Interventions*.

[103] Figulla HR, Franz M, Lauten A (2020). The History of Transcatheter Aortic Valve Implantation (TAVI)—a Personal View over 25 Years of development. *Cardiovascular Revascularization Medicine*.

[104] Thomas M, Schymik G, Walther T, Himbert D, Lefèvre T, Treede H (2010). Thirty-Day Results of the SAPIEN Aortic Bioprosthesis European Outcome (SOURCE) Registry: A European registry of transcatheter aortic valve implantation using the Edwards SAPIEN valve. *Circulation*.

[105] Smith CR, Leon MB, Mack MJ, Miller DC, Moses JW, Svensson LG (2011). Transcatheter versus surgical aortic-valve replacement in high-risk patients. *The New England Journal of Medicine*.

[106] Leon MB, Smith CR, Mack M, Miller DC, Moses JW, Svensson LG (2010). Transcatheter aortic-valve implantation for aortic stenosis in patients who cannot undergo surgery. *The New England Journal of Medicine*.

[107] Adams DH, Popma JJ, Reardon MJ (2014). Transcatheter aortic-valve replacement with a self-expanding prosthesis. *The New England Journal of Medicine*.

[108] Gleason TG, Reardon MJ, Popma JJ, Deeb GM, Yakubov SJ, Lee JS (2018). 5-Year Outcomes of Self-Expanding Transcatheter Versus Surgical Aortic Valve Replacement in High-Risk Patients. *Journal of the American College of Cardiology*.

[109] De Backer O, Götberg M, Ihlberg L, Packer E, Savontaus M, Nielsen NE (2016). Efficacy and safety of the Lotus Valve System for treatment of patients with severe aortic valve stenosis and intermediate surgical risk: Results from the Nordic Lotus-TAVR registry. *International Journal of Cardiology*.

[110] Feldman TE, Reardon MJ, Rajagopal V, Makkar RR, Bajwa TK, Kleiman NS (2018). Effect of Mechanically Expanded vs Self-Expanding Transcatheter Aortic Valve Replacement on Mortality and Major Adverse Clinical Events in High-Risk Patients with Aortic Stenosis: The REPRISE III Randomized Clinical Trial. *The Journal of the American Medical Association*.

[111] Kim W, Hengstenberg C, Hilker M, Kerber S, Schäfer U, Rudolph T (2018). The SAVI-TF Registry: 1-Year Outcomes of the European Post-Market Registry Using the ACURATE neo. *JACC: Cardiovascular Interventions*.

[112] Binder RK, Schäfer U, Kuck K, Wood DA, Moss R, Leipsic J (2013). Transcatheter Aortic Valve Replacement with a New Self-Expanding Transcatheter Heart Valve and Motorized Delivery System. *JACC: Cardiovascular Interventions*.

[113] Schäfer U, Kempfert J, Verheye S, Maisano F, Thiele H, Landt M (2020). Safety and Performance Outcomes of a Self-Expanding Transcatheter Aortic Heart Valve: The BIOVALVE Trials. *JACC: Cardiovascular Interventions*.

[114] Adams HSL, Prendergast B, Redwood S (2020). BIOVALVE: A New Self-Expanding Supra-Annular TAVR System. *JACC: Cardiovascular Interventions*.

[115] Naber CK, Pyxaras SA, Ince H, Frambach P, Colombo A, Butter C (2016). A multicentre European registry to evaluate the Direct Flow Medical transcatheter aortic valve system for the treatment of patients with severe aortic stenosis. *EuroIntervention*.

[116] Lefèvre T, Colombo A, Tchétché D, Latib A, Klugmann S, Fajadet J (2016). Prospective Multicenter Evaluation of the Direct Flow Medical Transcatheter Aortic Valve System: 12-Month Outcomes of the Evaluation of the Direct Flow Medical Percutaneous Aortic Valve 18F System for the Treatment of Patients With Severe Aortic Stenosis (DISCOVER) Study. *JACC: Cardiovascular Interventions*.

[117] Kische S, D’Ancona G, Agma HU, El-Achkar G, Dißmann M, Ortak J (2017). Trans-catheter aortic valve implantation with the direct flow medical prosthesis: Single center short-term clinical and echocardiographic outcomes. *Catheterization and Cardiovascular Interventions*.

[118] Vahanian A, Alfieri OR, Al-Attar N, Antunes MJ, Bax J, Cormier B (2008). Transcatheter valve implantation for patients with aortic stenosis: a position statement from the European Association of Cardio-Thoracic Surgery (EACTS) and the European Society of Cardiology (ESC), in collaboration with the European Association of Percutaneous Cardiovascular Interventions (EAPCI). *European Heart Journal*.

[119] Schymik G, Lefèvre T, Bartorelli AL, Rubino P, Treede H, Walther T (2015). European Experience with the second-Generation Edwards SAPIEN XT Transcatheter Heart Valve in Patients with Severe Aortic Stenosis: 1-year outcomes from the SOURCE XT Registry. *JACC: Cardiovascular Interventions*.

[120] Leon MB, Smith CR, Mack MJ, Makkar RR, Svensson LG, Kodali SK (2016). Transcatheter or Surgical Aortic-Valve Replacement in Intermediate-Risk Patients. *The New England Journal of Medicine*.

[121] Reardon MJ, Van Mieghem NM, Popma JJ, Kleiman NS, Søndergaard L, Mumtaz M (2017). Surgical or Transcatheter Aortic-Valve Replacement in Intermediate-Risk Patients. *The New England Journal of Medicine*.

[122] Voigtländer L, Seiffert M (2018). Expanding TAVI to Low and Intermediate Risk Patients. *Frontiers in Cardiovascular Medicine*.

[123] Vahanian A, Alfieri O, Andreotti F, Antunes MJ, Barón-Esquivias G, Baumgartner H (2012). The Joint Task Force on the Management of Valvular Heart Disease of the European Society of Cardiology (ESC); European Association for Cardio-Thoracic Surgery (EACTS), Guidelines on the management of valvular heart disease (version 2012). *European Heart Journal*.

[124] Nishimura RA, Otto CM, Bonow RO, Carabello BA, Erwin JP, Fleisher LA (2017). 2017 AHA/ACC Focused Update of the 2014 AHA/ACC Guideline for the Management of Patients With Valvular Heart Disease: A Report of the American College of Cardiology/American Heart Association Task Force on Clinical Practice Guidelines. *Journal of the American College of Cardiology*.

[125] Popma JJ, Deeb GM, Yakubov SJ, Mumtaz M, Gada H, O’Hair D (2019). Evolut Low Risk Trial Investigators. Transcatheter Aortic-Valve Replacement with a Self-Expanding Valve in Low-Risk Patients. *The New England Journal of Medicine*.

[126] Thyregod HGH, Ihlemann N, Jørgensen TH, Nissen H, Kjeldsen BJ, Petursson P (2019). Five-Year Clinical and Echocardiographic Outcomes from the Nordic Aortic Valve Intervention (NOTION) Randomized Clinical Trial in Lower Surgical Risk Patients. *Circulation*.

[127] Jørgensen TH, Thyregod HGH, Ihlemann N, Nissen H, Petursson P, Kjeldsen BJ (2021). Eight-year outcomes for patients with aortic valve stenosis at low surgical risk randomized to transcatheter vs. surgical aortic valve replacement. *European Heart Journal*.

[128] Siontis GCM, Overtchouk P, Cahill TJ, Modine T, Prendergast B, Praz F (2019). Transcatheter aortic valve implantation vs. surgical aortic valve replacement for treatment of symptomatic severe aortic stenosis: an updated meta-analysis. *European Heart Journal*.

[129] Fauvel C, Capoulade R, Durand E, Béziau DM, Schott J, Le Tourneau T (2020). Durability of transcatheter aortic valve implantation: a translational review. *Archives of Cardiovascular Diseases*.

[130] Pibarot P, Ternacle J, Jaber WA, Salaun E, Dahou A, Asch FM (2020). Structural Deterioration of Transcatheter Versus Surgical Aortic Valve Bioprostheses in the PARTNER-2 Trial. *Journal of the American College of Cardiology*.

[131] Blackman DJ, Saraf S, MacCarthy PA, Myat A, Anderson SG, Malkin CJ (2019). Long-Term Durability of Transcatheter Aortic Valve Prostheses. *Journal of the American College of Cardiology*.

[132] Søndergaard L, Ihlemann N, Capodanno D, Jørgensen TH, Nissen H, Kjeldsen BJ (2019). Durability of Transcatheter and Surgical Bioprosthetic Aortic Valves in Patients at Lower Surgical Risk. *Journal of the American College of Cardiology*.

[133] Ueyama H, Kuno T, Takagi H, Kobayashi A, Misumida N, Pinto DS (2021). Meta-Analysis Comparing Valve Durability among Different Transcatheter and Surgical Aortic Valve Bioprosthesis. *The American Journal of Cardiology*.

[134] Zghouzi M, Osman H, Ullah W, Suleiman A, Razvi P, Abdalrazzak M (2022). Safety and efficacy of transcatheter aortic valve implantation in stenotic bicuspid aortic valve compared to tricuspid aortic valve: a systematic review and meta-analysis. *Expert Review of Cardiovascular Therapy*.

[135] Xiong TY, Ali WB, Feng Y, Hayashida K, Jilaihawi H, Latib A (2022). Transcatheter aortic valve implantation in patients with bicuspid valve morphology: a roadmap towards standardization. *Nature Reviews Cardiology*.

[136] Faroux L, Junquera L, Mohammadi S, Del Val D, Muntané‐Carol G, Alperi A (2020). Femoral Versus Nonfemoral Subclavian/Carotid Arterial Access Route for Transcatheter Aortic Valve Replacement: a Systematic Review and Meta‐Analysis. *Journal of the American Heart Association*.

[137] Beurtheret S, Karam N, Resseguier N, Houel R, Modine T, Folliguet T (2019). Femoral Versus Nonfemoral Peripheral Access for Transcatheter Aortic Valve Replacement. *Journal of the American College of Cardiology*.

[138] D’Amore A, Luketich SK, Raffa GM, Olia S, Menallo G, Mazzola A (2018). Heart valve scaffold fabrication: Bioinspired control of macro-scale morphology, mechanics and micro-structure. *Biomaterials*.

[139] Mela P, D’Amore A (2020). In Situ Heart Valve Tissue Engineering: Is Scaffold Structural Biomimicry Overrated. *JACC: Basic to Translational Science*.

